# Increased expression of CDKN1A/p21 in HIV-1 controllers is correlated with upregulation of ZC3H12A/MCPIP1

**DOI:** 10.1186/s12977-020-00522-4

**Published:** 2020-07-02

**Authors:** Suwellen S. D. de Azevedo, Marcelo Ribeiro-Alves, Fernanda H. Côrtes, Edson Delatorre, Lucia Spangenberg, Hugo Naya, Leonardo N. Seito, Brenda Hoagland, Beatriz Grinsztejn, Valdilea G. Veloso, Mariza G. Morgado, Thiago Moreno L. Souza, Gonzalo Bello

**Affiliations:** 1 Laboratório de AIDS & Imunologia Molecular, Instituto Oswaldo Cruz–IOC, FIOCRUZ, Av. Brasil 4365, Rio de Janeiro, RJ 21045-900 Brazil; 2 Laboratório de Pesquisa Clínica em DST-AIDS, Instituto Nacional de Infectologia Evandro Chagas-INI, FIOCRUZ, Rio de Janeiro, Brazil; 3 Departamento de Biologia, Centro de Ciências Exatas, Naturais e da Saúde, Universidade Federal do Espírito Santo, Alegre, Brazil; 4 Unidad de Bioinformática, Institut Pasteur Montevideo, Montevideo, Uruguay; 5 Departamento de Informática y Ciencias de la Computación, Facultad de Ingeniería y Tecnologías, Universidad Católica del Uruguay, Montevideo, Uruguay; 6 Departamento de Producción Animal y Pasturas, Facultad de Agronomía, Universidad de la República, Montevideo, Uruguay; 7 Laboratório de Farmacologia Aplicada, Instituto de Tecnologia em Fármacos–Farmanguinhos FIOCRUZ, Rio de Janeiro, Brazil; 8 National Institute for Science and Technology on Innovation on Diseases of Neglected Populations (INCT/IDPN), FIOCRUZ, Center for Technological Development in Health-CDTS, Rio de Janeiro, Brazil; 9 Laboratório de Imunofarmacologia, Instituto Oswaldo Cruz–IOC, FIOCRUZ, Rio de Janeiro, Brazil

**Keywords:** HIV-1 controllers, Restriction factors, MCPIP1, p21, Immune activation

## Abstract

**Background:**

Some multifunctional cellular proteins, as the monocyte chemotactic protein-induced protein 1 (ZC3H12A/MCPIP1) and the cyclin-dependent kinase inhibitor CDKN1A/p21, are able to modulate the cellular susceptibility to the human immunodeficiency virus type 1 (HIV-1). Several studies showed that CDKN1A/p21 is expressed at high levels ex vivo in cells from individuals who naturally control HIV-1 replication (HIC) and a recent study supports a coordinate regulation of ZC3H12A/MCPIP1 and CDKN1A/p21 transcripts in a model of renal carcinoma cells. Here, we explored the potential associations between mRNA expression of ZC3H12A/MCPIP1 and CDKN1A/p21 in HIC sustaining undetectable (elite controllers–EC) or low (viremic controllers–VC) viral loads.

**Results:**

We found a selective upregulation of ZC3H12A/MCPIP1 and CDKN1A/p21 mRNA levels in PBMC from HIC compared with both ART–suppressed and HIV–negative control groups (P≤ 0.02) and higher MCPIP1 and p21 proteins levels in HIC than in HIV-1 negative subjects. There was a moderate positive correlation (r ≥ 0.57; P ≤ 0.014) between expressions of both transcripts in HIC and in HIC combined with control groups. We found positive correlations between the mRNA level of CDKN1A/p21 with activated CD4^+^ T cells levels in HIC (r ≥ 0.53; P ≤ 0.017) and between the mRNA levels of both CDKN1A/p21 (r = 0.74; P = 0.005) and ZC3H12A/MCPIP1 (r = 0.58; P = 0.040) with plasmatic levels of sCD14 in EC. Reanalysis of published transcriptomic data confirmed the positive association between ZC3H12A/MCPIP1 and CDKN1A/p21 mRNA levels in CD4^+^ T cells and monocytes from disparate cohorts of HIC and other HIV-positive control groups.

**Conclusions:**

These data show for the first time the simultaneous upregulation of ZC3H12A/MCPIP1 and CDKN1A/p21 transcripts in the setting of natural suppression of HIV-1 replication in vivo and the positive correlation of the expression of these cellular factors in disparate cohorts of HIV-positive individuals. The existence of a common regulatory pathway connecting ZC3H12A/MCPIP1 and CDKN1A/p21 could have a synergistic effect on HIV-1 replication control and pharmacological manipulation of these multifunctional host factors may open novel therapeutic perspectives to prevent HIV-1 replication and disease progression.

## Background

Among the individuals infected by the human immunodeficiency virus type 1 (HIV-1), a rare group called HIV controllers (HIC) suppress viral replication in absence of antiretroviral therapy, maintaining RNA viral loads (VL) below the limit of detection (LOD; elite controllers, EC) or at low levels (> LOD and < 2000 copies/ml; viremic controllers, VC). Natural control of HIV-1 replication is probably a multifactorial feature that involves different combinations of host and viral factors [1].

Some intrinsic host proteins, termed restriction factors (RF), are components of the innate immune response [2, 3] that have the ability to cause a significant reduction in viral infectivity by interacting directly with the pathogen. RF are, generally, interferon (IFN)-stimulated genes (ISGs) [4] and several has been shown to tackle different stages of HIV life cycle [3], including some classical RF such the Apolipoprotein B mRNA-Editing enzyme, Catalytic polypeptide-like (APOBEC3G), the Bone Stromal Tumor protein 2 (BST2)/Tetherin, the Sterile Alpha Motif domain and HD domain-containing protein 1 (SAMHD1) [2], and others more recently characterized like the Myxovirus resistance protein 2 (Mx2), the Interferon-inducible transmembrane family proteins (IFITM1-3 members), and Schlafen 11 (SLFN11) [3]. The mRNA levels of some RF including SAMHD1, Theterin, IFITM1, Mx2 and SLFN11 were described to be elevated in peripheral blood mononuclear cells (PBMC) or CD4^+^ T cells of HIC compared to antiretroviral (ART)-suppressed and/or HIV-uninfected individuals [5–9], although with contrasting findings across different HIC cohorts.

Others host multifunctional proteins, not recognized as classical RF, are also able to modulate the cellular susceptibility to HIV-1 infection. The cyclin-dependent kinase (CDK) inhibitor p21, encoded by the *CDKN1A* gene, modulates multiple relevant processes of the immune system, including proliferation of activated/memory T cells, macrophage activation and inflammation [10–17]. This protein also indirectly limits the HIV-1 replication in vitro in various cellular systems by blocking the biosynthesis of dNTPs required for viral reverse transcription and by inhibiting the CDK9 activity required for HIV-1 mRNA transcription [18–23]. Several studies described that CDKN1A/p21 is expressed at high levels ex vivo in CD4^+^ T cells from HICs [21, 24–26] and that p21 mRNA levels are correlated with CD4^+^ T cell activation in EC, but not in other HIV-infected groups [5]. These pieces of evidence suggest that the inducibility of CDKN1A/p21 to immune activation is a singular characteristic of EC and may contribute to the natural control of HIV-1 replication in vivo.

The monocyte chemotactic protein–induced protein 1 (MCPIP1), encoded by the *Zc3h12a* gene, is another newly discovered host multifunctional modulator of immune response with antiviral activity [27]. MCPIP1 plays a critical role in the regulation of the inflammatory response and immune homeostasis and also blocks HIV-1 replication in vitro by promoting the viral mRNA degradation through its RNase activity, particularly in quiescent CD4^+^ T cells [27, 28]. In activated CD4^+^ T cells, ZC3H12A/MCPIP1 is rapidly degraded [28] after its cleavage by the mucosa-associated lymphoid-tissue lymphoma-translocation 1 (MALT1) protein [29, 30]. In activated macrophage cells, by contrast, MCPIP1 transcripts are induced by TLR ligands and pro-inflammatory cytokines (mainly, TNF-α, IL-1β, and CCL2/MCP-1), and its expression stimulates a negative feedback loop that attenuates the inflammatory state by decreasing its fundamental mediators [27, 31]. The expression level of ZC3H12A/MCPIP1 in HIC was never described before.

Interestingly, a recent study of human renal carcinoma cell line (Caki-1 cells) revealed that ZC3H12A/MCPIP1 overexpression reduces the cellular growth by increasing the levels of CDKN1A/p21 transcripts, along with other proteins involved in cell cycle progression/arrest, supporting a coordinate regulation of ZC3H12A/MCPIP1 and CDKN1A/p21 in that cell-line [32]. This evidence prompted us to ask whether the expression of ZC3H12A/MCPIP1 could be elevated and positively correlated with CDKN1A/p21 in the setting of natural control of HIV-1 infection. To test this hypothesis, we quantified the ex vivo expression of ZC3H12A/MCPIP1 and CDKN1A/p21 mRNAs in PBMC from HIC, ART-suppressed, and HIV-uninfected individuals of disparate cohorts.

## Results

### The ZC3H12A/MCPIP1 and CDKN1A/p21 mRNA and protein expression levels are upregulated in PBMC from HIC

Twenty-nine HIV-1 positive (21 HIC and 8 ART-suppressed) and 10 HIV-negative individuals were included in this cross-sectional study. Most HIV-positive (59%) and HIV-negative (60%) individuals were females and all individuals displayed CD4^+^ T cells counts above 500 cells/μl (Table 1). Although the EC subgroup showed a higher proportion of females (77%) than other groups, the difference was not statistically significant (Additional file 1: Table S1).

**Table 1:** Main clinical and epidemiologic characteristics of individuals of this study

Characteristics	HIC (n = 21)		ART-suppressed (n = 10)	HIV-1 negative (n = 8)
	EC (n = 13)	VC (n = 8)		
Sex, no. (%)				
Female	10 (77)	3 (38)	4 (50)	6 (60)
Male	3 (23)	5 (62)	4 (50)	4 (40)
Age (years)^ab^	45 (39–60)	44 (39–47)	47 (38–53)	47 (36–51)
Study point				
Time since HIV-1 diagnosis (years)^b^	9 (5.5–15)	12.5 (7–16)	NA	–
CD4^+^ T cell (cells/μl)^b^	1027 (834–1255)	664 (563–1228)	889 (678–1097)	1043 (784–1581)
Plasma HIV RNA (copies/ml)^b^	< 50	641 (327–915)	< 40	–
CD4/CD8 ratio^b^	1.33 (1.24–1.61)	0.91 (0.67–1.23)	1.06 (0.73–1.5)	1.69 (1.62–2.00)

*HIC* HIV controllers, *ART* antiretroviral therapy, *EC* elite controllers, *VC* viremic controllers, *NA* not available

^a^Age at study point; ^b^Interquartile ranges are shown in parenthesis

**Fig. 1:**
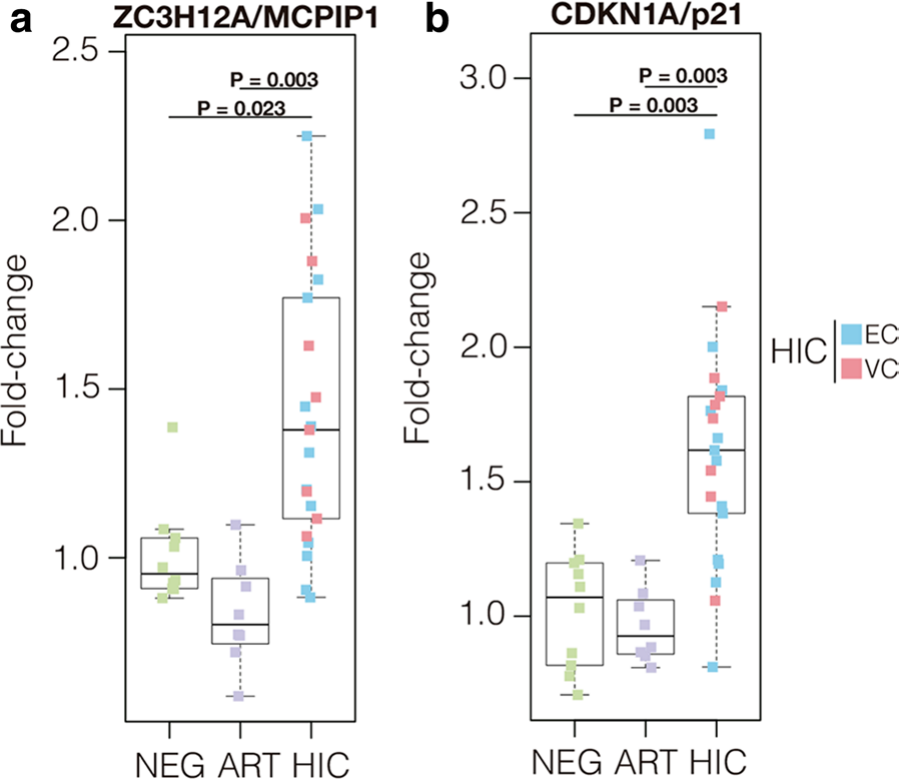
ZC3H12A/MCPIP1 and CDKN1A/p21 mRNA levels are upregulated in PBMC from HIC. Boxplots represent the interquartile and sample median (central solid black line) of the relative changes (fold-change values relative to the mean of HIV-1-uninfected (NEG) subjects) of ZC3H12A/MCPIP1 **a** and CDKN1A/p21 **b** expression comparing NEG and ART-suppressed subjects (ART) with HIV controllers (HIC). P-values < 0.05 were considered statistically significant

**Fig. 2:**
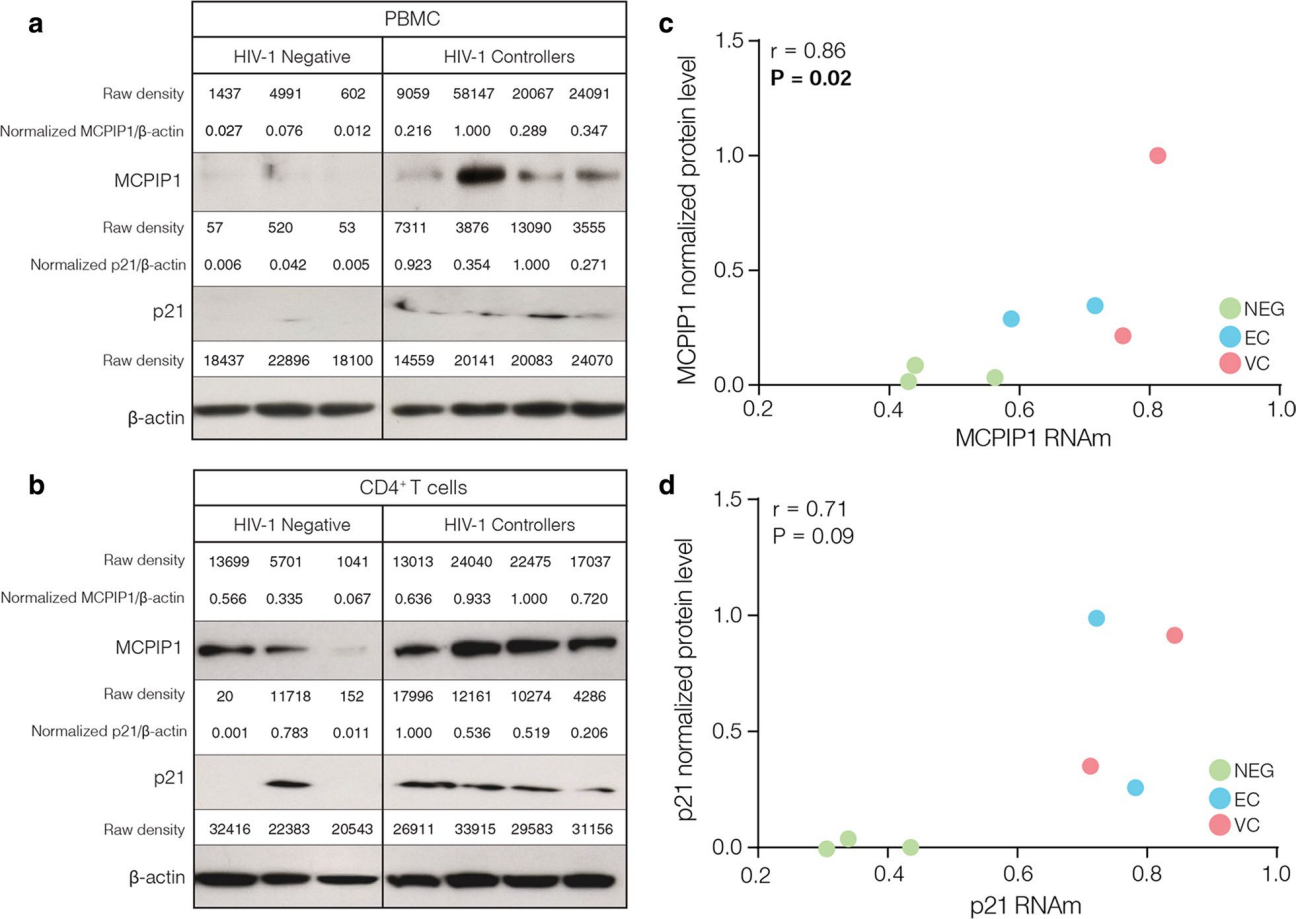
MCPIP1 and p21 protein levels are increased in HIC. The MCPIP1 and p21 proteins expression in PBMC **a** and CD4^+^ T cells **b** from HIV-negative and HIV controllers, was determined by western blot. The quantified MCPIP1 and p21 protein expression levels were normalized with β-actin protein levels and immunoblotting bands were quantified with ImageJ64 software. Correlation between normalized proteins and mRNA levels is shown to MCPIP1 **c** and p21 **d**. Correlation coefficients (Spearman’s ρ) are shown in the upper right corner of each graph. P-values < 0.05 were considered statistically significant

Analysis of the expression of multifunctional genes revealed a significant (*P* < 0.05) upregulation of both ZC3H12A/MCPIP1 (Fig. 1a) and CDKN1A/p21 (Fig. 1b) transcripts in PBMC from HIC compared to cells from ART-suppressed (1.68-fold increase and 1.63-fold increase, respectively) and HIV-negative (1.37-fold increase and 1.55-fold increase, respectively) individuals (Fig. 1). Analysis of the mRNA levels of several antiretroviral RF, by contrast, failed to reveal significant differences between the HIC and control groups, with the only exception of significant upregulation of IFITM1 in HIC respect to the HIV-negative group (Additional file 1: Figure S1). We also measured the expression level of MCPIP1 and CDKN1A proteins in PBMC and CD4^+^ T cells from a subset of four HIC (two EC and two VC) and three HIV-1 negative subjects (Additional file 1: Figure S2). Western blot analyses demonstrated that the median expression levels of MCPIP1 and p21 proteins in PBMC and CD4^+^ T cells from HIC were higher than in HIV-negative individuals (Fig. 2a, b). Moreover, we observed strong positive correlation between the mRNA and protein expression of ZC3H12A/MCPIP1 (r = 0.86, P = 0.024) and CDKN1A/p21 (r = 0.71, P = 0.09) in PBMC (Fig. 2c, d). These results support a selective upregulation of both ZC3H12A/MCPIP1 and CDKN1A/p21 mRNA levels in PBMC from our HIC cohort and further suggest that transcripts levels are correlated with levels of protein expression.

### Positive correlation of ZC3H12A/MCPIP1 and CDKN1A/p21 mRNA expression in PBMC

**Fig. 3:**
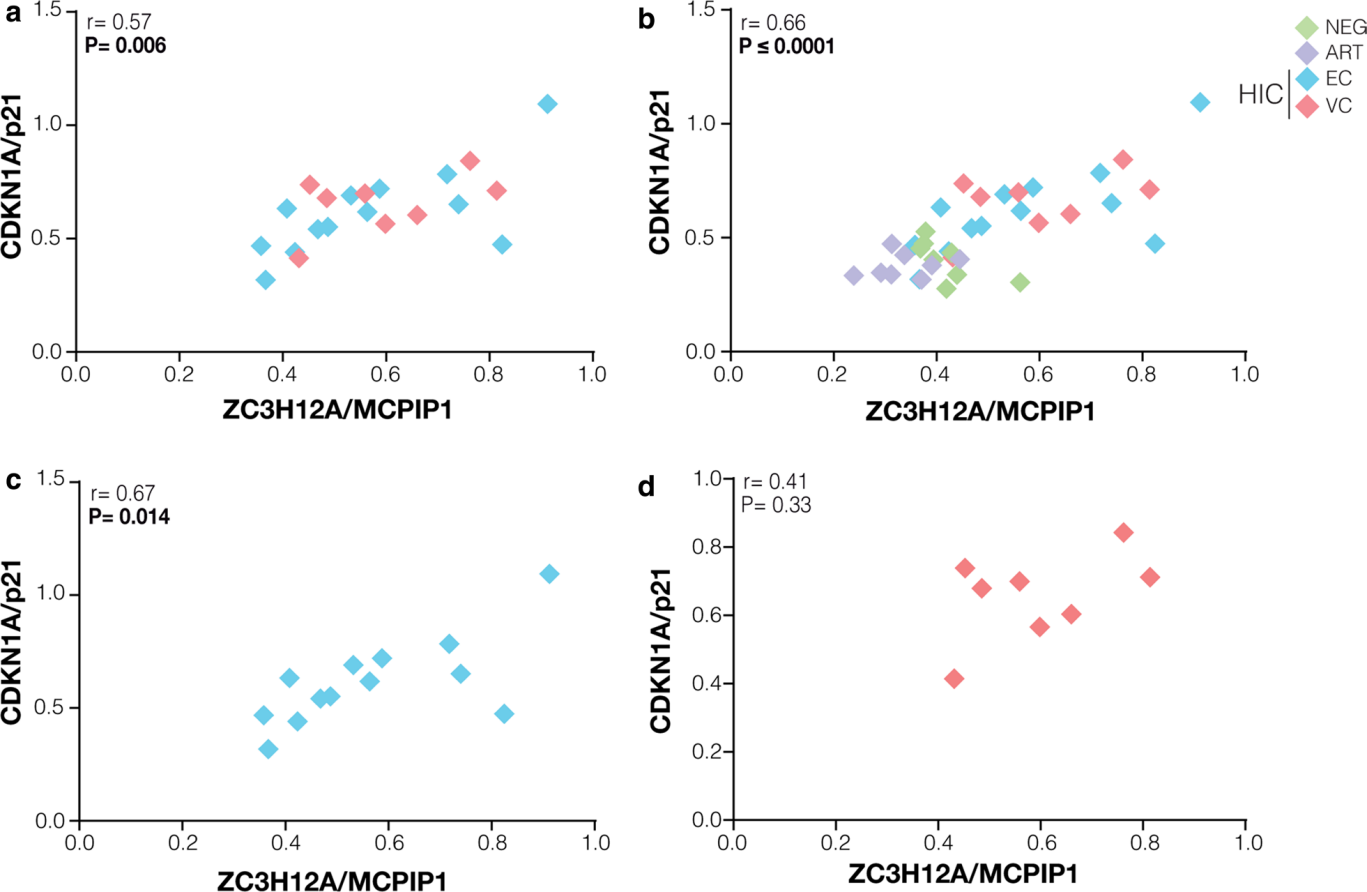
CDKN1A/p21 and ZC3H12A/MCPIP1 mRNA levels in PBMC from HIC are positively correlated. The CDKN1A/p21 and ZC3H12A/MCPIP1 normalized expression correlations were calculated considering HIC **a**, all groups **b**, elite controllers **c**, and viremic controllers **d**. The points’ colors indicate the patient group, accordingly to the legend. Correlation coefficients (Spearman’s ρ) are shown in the upper right corner of each graph. P-values < 0.05 were considered statistically significant

A recent study supports a coordinate regulation of ZC3H12A/MCPIP1 and CDKN1A/p21 in a human renal carcinoma cell line [32]. Consistent with this finding, we observed a significant positive correlation between the mRNA expression of ZC3H12A/MCPIP1 and CDKN1A/p21 in PBMC from HIC (r = 0.57; P = 0.006) (Fig. 3a). Positive correlations trends were also maintained when HIC were combined with control groups (r = 0.66; P ≤ 0.0001) (Fig. 3b), when EC (r = 0.67; P = 0.014) and VC (r = 0.41; P = 0.33) sub-groups were analyzed separately (Fig. 3c, d), and when individuals were subdivided by sex (Additional file 1: Figure S3). By contrast, no significant correlations were observed between the mRNA expression of ZC3H12A/MCPIP1 or CDKN1A/p21 and the RF analyzed, with the only exception of a significant negative correlation between expression of those multifunctional genes and APOBEC3G in HIC (Additional file 1: Figure S4). These results demonstrate a coordinated mRNA expression of ZC3H12A/MCPIP1 and CDKN1A/p21 in PBMC from HIC that was not positive correlated with expression of classical RF. These results further suggest that such association is probably not an exclusive characteristic of HIC, but can also be detected in PBMC from others groups of HIV-infected and uninfected individuals.

### MCPIP1 and p21 mRNA expression is positive correlated with immune activation in HIC

**Fig. 4:**
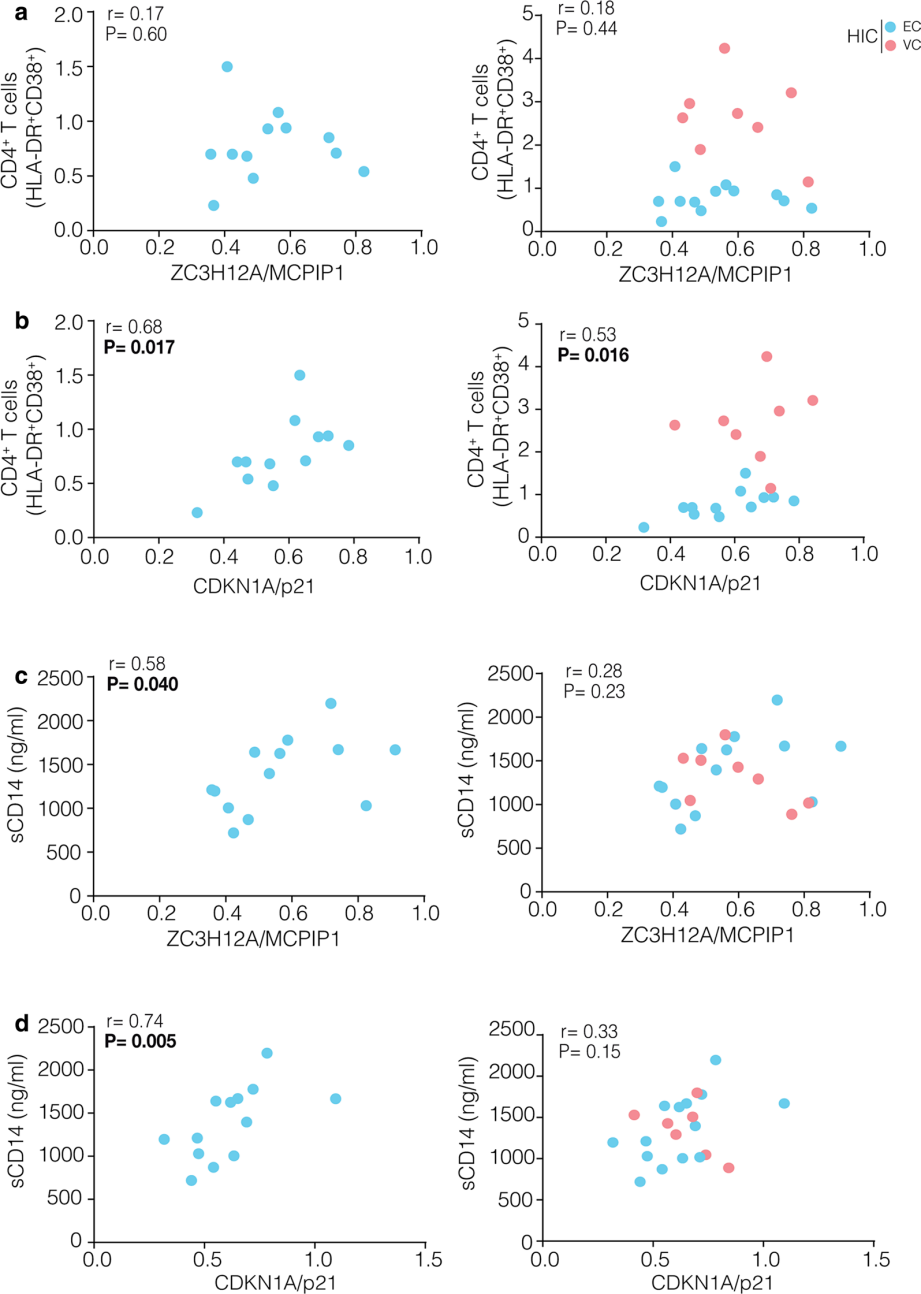
CDKN1A/p21 transcripts are positively correlated with CD4^+^ T cell and monocyte activation while ZC3H12A/MCPIP1transcripts are positively correlated only with monocyte activation in EC. The correlations were made evaluating the relationship between activated CD4^+^ T cells **a**, **b** or sCD14 levels **c**, **d** with the normalized expression of p21 and MCPIP1 for EC and HIC groups. The points’ colors present in each graph indicate the groups present according to the legend. Correlations coefficient (Spearman’s ρ) is shown in the upper left corner of each graph

A previous study showed that CDKN1A/p21 mRNA levels are positive correlated with CD4^+^ T cell activation in HIC [5, 21]. To explore the potential relationship of CDKN1A/p21 or ZC3H12A/MCPIP1 expression with immune activation, we measured the frequency of HLA-DR^+^CD38^+^ phenotype on CD4^+^ and CD8^+^ T cells (T cell activation) and plasma levels of sCD14 (monocyte activation) in our cohort. Frequencies of activated CD4^+^ T cell populations in VC and ART-suppressed subjects were higher than in EC (*P* < 0.0001) and HIV-negative (*P* = 0.0002) individuals (Additional file 1: Figure S5A). The VC subgroup also had significantly higher frequencies of activated CD8^+^ T cell than EC (*P* = 0.0007) and control groups (*P* ≤ 0.006) (Additional file 1: Figure S5B). The median concentration of sCD14 in plasma was not significantly different across the groups (Additional file 1: Figure S5C). No significant correlations between mRNA levels of ZC3H12A/MCPIP1 and CD4^+^ T cell (Fig. 4a) or CD8^+^ T cell (Additional file 1: Figure S6A) activation were observed for HIC. The mRNA levels of CDKN1A/p21 were positively associated with activated CD4^+^ T cells levels in HIC (r = 0.53; *P* = 0.016) (Fig. 4b); but not with activated CD8^+^ T cell levels (Additional file 1: Figure S6B). Levels of sCD14 were positively correlated with both ZC3H12A/MCPIP1 (r = 0.58; *P* = 0.040) and CDKN1A/p21 (r = 0.74; *P* = 0.005) mRNA levels only in the EC subset (Fig. 4c, d). No significant correlations between mRNA levels of MCPIP1/p21 and CD4^+^/CD8^+^ T cell activation or sCD14 levels were observed when ART-suppressed and HIV-negative individuals were included (Additional file 1: Figure S7). Multivariate analysis showed that the upregulation of ZC3H12A/MCPIP1 was positively associated with the increase of CDKN1A/p21 expression in HIC (1.44-fold increase; *P* = 0.0035) (Additional file 1: Figure S8A). The frequency of activated CD4^+^ T cells also was positively associated with the increase of CDKN1A/p21 expression in both EC and VC (1.48-fold increase; *P* = 0.0116), although this increase of the CDKN1A/p21 expression was down-regulated by the increase of activated CD4^+^ T cells in VC when compared to EC (1.30-fold decrease by an increase of 1% CD4^+^HLA-DR^+^CD38^+^ T cells; *P* = 0.0284) (Additional file 1: Figure S8B). Overall, the model was highly significant (*P* = 0.003) and could explain as much as 70% (R^2^ = 0.492) of CDKN1A/p21 expression. These results suggest that expression of CDKN1A/p21 in PBMC from HIC may be modulated by both ZC3H12A/MCPIP1 expression and CD4^+^ T cell activation.

### Positive correlation between ZC3H12A/MCPIP1 and CDKN1A/p21 mRNA expression in CD4^+^ T cells and primary monocytes from disparate cohorts of HIV-infected and uninfected subjects

To further validate our observations, we analyzed the expression profiles of ZC3H12A/MCPIP1 and CDKN1A/p21 in HLA-DR^−^ CD4^+^ T cells [33], total CD4^+^ T cells [34, 35], HIV-specific CD4^+^ T cells [36], total CD8^+^ T cells [35] and primary monocytes [37, 38] previously reported for other cohorts (Additional file 1: Table S2). All selected studies include data of HIC, with the only exception of the studies of monocytes, and different control groups including: ART-suppressed, ART-non-suppressed, untreated non-controllers typical progressors (NC-TP), untreated non-controllers long-term nonprogressors (NC-LTNP), and/or HIV-negative individuals [33–38]. Pairwise comparisons revealed modest (logFC ≤ 2.5), but significant (*P* < 0.05), upregulation of ZC3H12A/MCPIP1 and CDKN1A/p21 in HIC compared to HIV-negative individuals from all cohorts (Additional file 1: Table S2). We also detect significant upregulation of CDKN1A/p21 isoform in HLA-DR^−^ CD4^+^ T cells from EC respect to ART-suppressed individuals and significant upregulation of both CDKN1A/p21 and ZC3H12A/MCPIP1 in HIV-specific CD4^+^ T cells from HIC respect to NC-TP and ART-suppressed individuals (Additional file 1: Table S2). These results suggest that upregulation of CDKN1A/p21 and ZC3H12A/MCPIP1 in HIC respect to other HIV-infected groups is a characteristic common to several, but not all, cohorts.

**Fig. 5:**
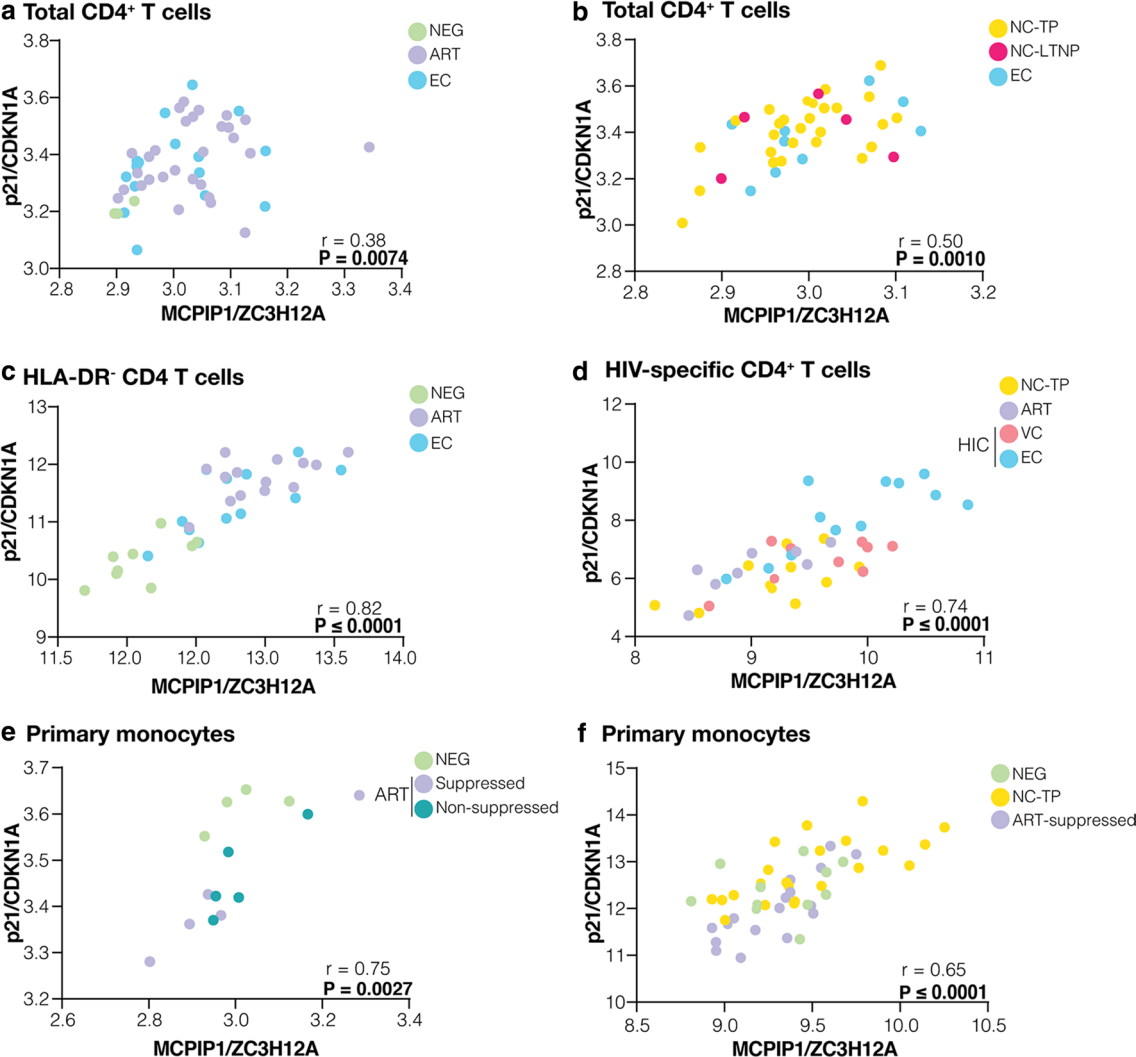
CDKN1A/p21 and ZC3H12A/MCPIP1 mRNA levels are positively correlated in total, HLA-DR^−^ and HIV-specific CD4^+^ T cells and primary monocytes. The ZC3H12A/MCPIP1 and isoform of CDKN1A/p21 (probe ILMN_1784602) normalized expression correlations were performed with data from previous studies in **a**, **b** total CD4^+^ T cells (GSE18233, GSE28128), **c** HLA-DR- CD4^+^ T cells (GSE23879), **d** HIV-specific CD4^+^ T cells and **e**, **f** primary monocytes (GSE52900, GSE18464) considering all groups (EC, VC, NC-TP, NC-LTNP, ART-suppressed, ART-non-suppressed, and HIV-negative subjects). The points’ colors indicate the patient group, accordingly to the legend. Correlation coefficients (Spearman’s ρ) are shown in the bottom right corner of each graph. P-values < 0.05 were considered statistically significant

Analyses of published PBMC transcriptomic data confirm a significant positive correlation between the mRNA expression of ZC3H12A/MCPIP1 and CDKN1A/p21 (r ≥ 0.38; P ≤ 0.0074) in all cohorts analyzed for CD4^+^ T cells and primary monocytes cell types (Fig. 5), but not for CD8^+^ T cells (Additional file 1: Figure S9). Overall, the most abundant CDKN1A isoform demonstrated a much better significant positive correlation with ZC3H12A/MCPIP1 mRNA expression (Fig. 5) than the less abundant CDKN1A.1 isoform (r ≥ 0.30; P ≤ 0.45) (Additional file 1: Figure S10). Correlations coefficients between expression of ZC3H12A/MCPIP1 and CDKN1A/p21 in non-activated HLA-DR^−^ CD4 T cells (r = 0.82), HIV-specific CD4^+^ T cells (r = 0.74) and monocytes (r ≥ 0.65) were higher than in total CD4^+^ T cells (0.27 ≥ r ≥ 0.50). Significant positive correlations between the mRNA expression of ZC3H12A/MCPIP1 and CDKN1A/p21 in CD4^+^ T cells and primary monocytes were observed across studies combining different groups including: HIC, ART-suppressed and HIV-negative individuals (Fig. 5a, c); EC and non-controllers individuals (Fig. 5b); HIC, ART-suppressed individuals and non-controllers individuals (Fig. 5d); ART-suppressed, ART-non-suppressed and HIV-negative individuals (Fig. 5e); and ART-suppressed, non-controllers and HIV-negative individuals (Fig. 5f). Positive correlations were also observed when different HIV-positive groups were analyzed separately (Additional file 1: Figure S11). These data clearly support that coordinated regulation of mRNA expression of the ZC3H12A/MCPIP1 and CDKN1A/p21 seems to be a common characteristic of CD4^+^ T cells and monocytes subpopulations from different HIV-positive groups.

## Discussion

In this study, we described a positive correlation of ZC3H12A/MCPIP1 and CDKN1A/p21 mRNA expression in PBMC, CD4^+^ T cells and monocytes from HIV-infected and-uninfected subjects and we further detected that the mRNA levels of ZC3H12A/MCPIP1 and CDKN1A/p21 were significantly increased in PBMC of HIC compared to cells of HIV-negative and ART-suppressed individuals. We also observed higher MCPIP1 and p21 protein levels in PBMC and CD4^+^ T cells from HIC when compared with HIV-negative subjects.

Analyses of mRNA expression data in PBMC, CD4^+^ T cells and primary monocytes obtained from different cohorts of HIV–infected subjects described here and in previous studies clearly support a coordinated expression of ZC3H12A/MCPIP1 and at least one isoform of CDKN1A/p21, consistent with findings on human carcinoma cell line [32]. Of note, the ZC3H12A/MCPIP1 and CDKN1A/p21 mRNA expression seems to be much better correlated in HLA-DR^−^ CD4^+^ T cells than in total CD4^+^ T cells, indicating that activation of CD4^+^ T cells might partially disrupt that correlation. Consistent with this hypothesis, we observed that levels of CDKN1A/p21 mRNA, but not ZC3H12A/MCPIP1, were positively correlated with CD4^+^ T cell activation in HIC. We speculate that expression of CDKN1A/p21 in CD4^+^ T cells could be modulated by ZC3H12A/MCPIP1 and by stimuli that activate the TCR. Because ZC3H12A/MCPIP1 acts as a broad suppressor of the biogenesis pathway of both cellular [39] and viral miRNA [40], it might enhance the expression of CDKN1A/p21 by downregulating miRNAs that target its mRNA, like let-7c miRNA [41].

While elevated mRNA expression of CDKN1A/p21 in PBMC and CD4^+^ T cells of HIC had already been previously described in others cohorts [5, 21, 24–26], this is the first study to show simultaneous upregulation of ZC3H12A/MCPIP1 in HIC. The increased transcripts levels were mirrored by the protein products of these genes in PBMC, in accordance with previous studies [21, 42]. One limitation of our study is the impossibility of assigning which cell(s) population(s) has increased expression of ZC3H12A/MCPIP1 and CDKN1A/p21 in our cohort of HIC as the expression profile of these multifunctional proteins and RF may vary between different PBMC sub-populations [8]. Another limitation of our study is that the PBMC composition may vary among individuals depending on HIV-infection status, virologic control and immune deregulation, thus making comparison between different groups of HIV-positive and -negative individuals more difficult to interpret. Future studies comparing the expression levels of these multifunctional proteins in specific cell types might better decipher the mechanisms of host factors regulation in the setting of natural control of HIV-1 infection.

The reanalysis of previously reported expression profiles in specific PBMC subpopulations provide some conflicting results. Consistent with our study, we detect a modest, but significant, upregulation of both ZC3H12A/MCPIP1 and CDKN1A/p21 in CD4^+^ T cells of HIC compared to HIV-negative individuals from different cohorts [33, 34] as well as in HIV-specific CD4^+^ T cells of HIC respect to non-controllers and ART-suppressed individuals [36]. We also detected significant upregulation of CDKN1A/p21, but not ZC3H12A/MCPIP1, in HLA-DR^−^ CD4^+^ T cells from HIC respect to ART-suppressed individuals [33]. Finally, we failed to detect significant upregulation of these genes in total CD4^+^ T cells from HIC respect to HIV-infected control groups [34, 35]. These findings support that expression levels of ZC3H12A/MCPIP1 and CDKN1A/p21 might greatly vary among distinct PBMC sub-populations and HIV-infected individuals [43] and that the upregulation of those multifunctional proteins might not be a host signature common to all HIC.

Increased expression of some host RF has been previously observed in CD4^+^ T cells (i.e., SAMHD1, SLFN11 and IFITM1) [5, 7, 8] and PBMC (i.e., Mx1, Mx2, Tetherin and SLFN11) [6, 9] from some HIC. Although a few HIC from our cohort displayed mRNA levels of SAMHD1 and/or SLFN11 well above the normal range (Additional file 1: Figure S1), none of those RF were significantly upregulated in PBMC of our HIC cohort, with the only exception of IFITM1. Most ISGs are upregulated in the chronic phase of HIV-1 infection in viremic untreated patients [34, 44, 45] and their expression is positively correlated with the percentage of activated T cells and negatively correlated with CD4^+^ T cell counts [34, 44–47]. It is possible that residual or low-level viremia detected in HIC might not be enough to induce a generalized upregulation of ISGs during chronic infection [34]. In addition, ZC3H12A/MCPIP1 [48, 49] and CDKN1A/p21 [16] negatively regulate the NF-κB cascade and their upregulation may also contribute to limit the chronic upregulation of ISGs in our HIC cohort. While most RF are mainly induced by IFN type I, IFITM1 can also be induced by IFN type II [50] indicating that another pathway may have stimulated its expression in our HIC cohort.

The particular set of host RF and multifunctional proteins that are selectively upregulated clearly vary across disparate HIC cohorts. While one study detected upregulation of SAMHD1 mRNA in PBMCs from HIC compared to HIV-negative individuals [7], other study failed to detect significant differences in SAMHD1 expression between those groups [51]. Contrasting findings could be observed even among studies that analyzed specific cell types. Some works detected increased expression of CDKN1A/p21 [21, 24] or IFITM1 [8] in CD4^+^ T cells of HIC compared to HIV-negative individuals, but other study failed to detected significant upregulation of those proteins in CD4^+^ T cells of HIC [5]. Thus, cell types are not the only source of variation across studies of HIC. We propose that the genetic and immunologic heterogeneity of HIC individuals and HIV-infected control groups combined with the reduced size of most HIC cohorts might explain the contrasting findings across studies [5–9, 51].

Selective upregulation of ZC3H12A/MCPIP1 and CDKN1A/p21 in CD4^+^ T, macrophages and/or dendritic cells may have a synergistic antiviral effect and directly limit HIV-1 replication by (1) reducing the reverse transcription and chromosomal integration of HIV-1 in quiescent cells and thus limiting the size of the latent proviral reservoir [18–20, 52–54]; (2) restricting HIV-1 LTR transcription [48, 49, 55, 56]; and (3) degrading viral mRNA and miRNA [28, 39, 40, 57]. Whether upregulation of these multifunctional proteins directly contributes to reduce the susceptibility of CD4^+^ T cells and/or macrophages to HIV-1 infection in vivo, however, has been more difficult to prove. Chen et al. [21] demonstrate that elevated levels of CDKN1A/p21 in CD4^+^ T cells from HIC functionally contribute to the resistance of cells to HIV-1 infection in vitro. Saez-Cirion et al. [24] also detected high levels of CDKN1A/p21 in CD4^+^ T cells from HIC ex vivo, but found no evidence of a direct role of this protein in the reduced susceptibility of CD4^+^ T cells and macrophages to HIV-1 infection in vitro. Future studies of the susceptibility of PBMC of HIC to HIV-1 infection in vitro should analyze the potential role of both CDKN1A/p21 and ZC3H12A/MCPIP1 simultaneously.

Selective upregulation of ZC3H12A/MCPIP1 and CDKN1A/p21 may also play a crucial role in the control of HIV-1 infection by indirect mechanisms. HIV-1 preferentially infects the HIV-specific CD4^+^ memory T cells in vivo [58] and reduces their life span [59], compromising the generation of effective immune responses to the virus. Notably, our analysis of mRNA expression data in HIV-specific CD4^+^ T cells from a previous study [36] revealed increased levels of both ZC3H12A/MCPIP1 and CDKN1A/p21 transcripts in cells from HIC respect to other HIV-infected control groups. This may limit the seeding and size of the viral reservoir and further assist to preserve the specific CD4^+^ T response against HIV-1 in HIC. Because CDKN1A/p21 and ZC3H12A/MCPIP1 are negative regulators of the proliferation of activated/memory T cells [10, 13, 14, 29] and of macrophage-mediated inflammatory responses [15–17, 60, 61], upregulation of these proteins may also indirectly limit HIV-1 replication and further prevent CD4^+^ T cells decline by reducing chronic IFN-I signaling, generalized inflammation and over-activation of the immune system [10, 13–17, 60–63].

Our results confirm previous observations that levels of CDKN1A/p21 mRNA are positively correlated with CD4^+^ T cell activation in HIC [5] and further support a positive correlation between CDKN1A/p21 and ZC3H12A/MCPIP1 mRNA with monocyte activation in EC. Because induction of ZC3H12A/MCPIP1 mRNA upon CD4^+^ T cell activation is more ephemeral (< 12 h) than upon stimulation of macrophages (≥ 24 h) [60, 61, 64, 65], a direct correlation between ZC3H12A/MCPIP1 mRNA and CD4^+^ T cell activation could be more difficult to be detected. Increased expression of CDKN1A/p21 and ZC3H12A/MCPIP1 associated with T cell and/or monocyte activation may be a distinctive homoeostatic response to limit the deleterious effects of aberrant chronic immune activation and inflammation-driven by HIV-1 infection. Notably, similar correlations were not observed in other HIV-infected subjects in this or in previous studies [21], suggesting that this homoeostatic response could be a unique characteristic of some HIC/EC.

Transcript levels of RF here analyzed were not significantly correlated with T cell activation or sCD14 in HIC, with the only exception of a negative correlation between APOBEC3G mRNA and sCD14 levels in EC (r = − 0.73. P = 0.006; data not shown). Surprisingly, transcripts levels of APOBEC3G were also negatively correlated with ZC3H12A/MCPIP1 and CDKN1A/p21 mRNA levels in EC. One possible explanation for these negative correlations lies in the interaction of APOBEC3G, ZC3H12A/MCPIP1, and CDKN1A/p21 with the product of an important monocyte differentiation gene, the Kruppel-like factor 4 (KLF4). The expression of KLF4 in human macrophages is induced after IFN-γ, LPS, or TNF-α stimulus [66], mediating the proinflammatory signaling and the direct transcriptional regulation of CD14 in vitro [67] and also inducing expression of both ZC3H12A/MCPIP1 [68] and CDKN1A/p21 [69, 70]. Interestingly, APOBEC3G binds to the 3′-UTR of KLF4 mRNA and results in the reduction of its expression [71]. Thus, lower levels of APOBEC3G mRNA may be associated with an upregulation of KLF4 that in turn induce higher levels of sCD14, ZC3H12A/MCPIP1 and CDKN1A/p21 mRNA in EC.

## Conclusions

In summary, our data confirm the high levels of CDKN1A/p21 expression and show for the first-time the concurrent upregulation of ZC3H12A/MCPIP1 in HIC, suggesting a possible synergistic effect of both innate host multifunctional proteins on natural suppression of HIV-1 replication in vivo. Moreover, we found a positive correlation between CDKN1A/p21 and ZC3H12A/MCPIP1 transcripts in PBMC, CD4^+^ T cells, and monocytes from different HIV-infected cohorts as well as positive correlation between expression of those multifunctional proteins and activation of CD4^+^ T cells and/or macrophages. These findings point that pharmacological manipulation of CDKN1A/p21 and ZC3H12A/MCPIP1 may open novel therapeutic perspectives to reduce HIV-1 replication and to attenuate HIV-associated inflammation and immune activation in vivo.

## Methods

### Study subjects

We analyzed a cohort of 21 HIC subjects followed-up at the Instituto Nacional de Infectologia Evandro Chagas (INI) in Rio de Janeiro, Brazil. All HIC maintained RNA VL of < 2000 copies/ml without antiretroviral therapy for at least 5 years and were subdivided in two sub-groups: EC (*n* = 13) when most (≥ 70%) plasma VL determinations were below the limit of detection (LOD), and VC (*n* = 8) when most (≥ 70%) VL determinations were > LOD and < 2000 copies/ml. The limit of detection of plasma VL determinations varied over the follow-up period in according to the Brazilian Ministry of Health guidelines, with methodologies being updated overtime to improve sensitivity: Nuclisens HIV-1 RNA QT assay (Organon Teknika, Durham, NC, limit of detection: 80 copies/mL) from 1999 to 2007; the Versant HIV-1 3.0 RNA assay (bDNA 3.0, Siemens, Tarrytown, NY, limit of detection: 50 copies/mL) from 2007 to 2013; and the Abbott RealTime HIV-1 assay (Abbott Laboratories, Wiesbaden, Germany, limit of detection: 40 copies/mL) from 2013 to until today. Virological and immunological characteristics of these subjects were described in detail in previous studies [72, 73]. Two groups of ART-suppressed subjects (ART, *n* = 8) and healthy HIV-1-uninfected subjects (NEG, *n* = 10) were used as controls.

### mRNA gene-expression analysis

Total RNA was extracted from 1 × 10^7^ PBMC using RNeasy mini kit (Qiagen, Hilden, North Rhine-Westphalia, Germany) in which buffer RLT was supplemented with β-mercaptoethanol and displaced on-column DNase treatment using a Qiagen RNase-Free DNase Set (Qiagen, Hilden, North Rhine-Westphalia, Germany) according to manufacturer’s instruction. Total RNA yield and quality were determined using NanoDrop^®^ 8000 spectrophotometer and an Agilent^®^ 2100 Bioanalyzer. Only samples with an RNA integrity number (RIN) greater than 8.0 were used. Purified RNA (1 μg) was reverse-transcribed to cDNA using RT^2^ First Strand Kit (Qiagen, Hilden, North Rhine-Westphalia, Germany). The cDNA was mixed with RT^2^SYBR Green/ROX qPCR Master Mix (Qiagen, Hilden, North Rhine-Westphalia, Germany) and the mixture was added into customized RT^2^RNA PCR Array (Qiagen, Hilden, North Rhine-Westphalia, Germany) to measure the mRNA expression of 10 cellular target genes (APOBEC3G, SAMHD1, Tetherin, Mx1, Mx2, SLFN11, IFITM1, IFITM3, ZC3H12A/MCPIP1, and CDKN1A/p21) besides three housekeeping genes (GAPDH, β-actin, and RNase-P), according to manufacturer’s instructions. Values of the crossing point at the maximum of the second derivative of the four-parameters fitted sigmoid curve second derivative, Cp, was determined for each sample. The efficiency of each amplification reaction was calculated as the ratio between the fluorescence of the cycle of quantification and fluorescence of the cycle immediately preceding that. Genes used in the normalization among samples were selected by the geNorm method [74]. Data were expressed as fold-changes in mRNA abundance calculated as the normalized gene expression in any test sample divided by the mean normalized gene expression in the control HIV-negative group.

### Western blotting

We selected a subset of the eight cryopreserved PBMC samples (two elite controller, two viremic controllers and four healthy HIV-1-uninfected subjects) chosen based on a range of mRNA expression and availability of the biorepository for quantification of protein expression in PBMC and CD4^+^ T cells. CD4^+^ T cells were enriched from cryopreserved PBMCs using the EasySep Human CD4^+^ T Cell enrichment magnetic kit (StemCell Technologies, Vancouver, BC, Canada), according to the manufacturer’s instructions. The whole cell lysates were prepared from the 2 × 10^6^ PBMC and CD4^+^ T cells and lysed with RIPA buffer (Tris–HCl 50 mM, pH 8.0, 150 mM NaCl, 1% NP40, 0.1% SDS, 0.5% Sodium Deoxycholate, 1 mM PMSF, and protease inhibitor cocktail). Supernatants were recovered after 14,000 rpm centrifugation (10 min, 4 °C) and protein concentrations were determined according to the Lowry method (Bio-Rad DC protein assay kit). 30 µg of proteins from each sample were resolved in 10% SDS-PAGE, and proteins were electrotransferred to polyvinylidene difluoride (PVDF) membranes. Unspecific binding sites were blocked with 5% skimmed milk in Tris-buffered saline containing 0.1% Tween 20 (TBST) for 2h under agitation, before the probing with anti-p21 antibody (Cell Signaling Technology, Danvers, Massachusetts, EUA) overnight at 4° C. Unbound primary antibody was washed out with TBST before incubation with secondary anti- rabbit antibody (Cell Signaling Technology, Danvers, Massachusetts, EUA) for 1h. After that, the membranes were washed 3× with TBST and 2x with Tris-buffered saline (TBS) 1x before development by Enhanced Chemiluminescence (Thermo Scientific SuperSignal West Dura ECL) on Hyperfilm (Amersham Hyperfilm ECL). The second probing proceeded with anti-MCPIP1 antibody (Proteintech, Rosemont, IL 60,018, USA) incubation for 1.5h at room temperature (accordingly to the manufacturer), followed by the same steps of washing and incubation with secondary anti-rabbit antibody before ECL development. Finally, the membranes were probed and developed (Amersham ECL Prime) for β-Actin (Cell Signaling Technology, Danvers, Massachusetts, EUA), and the immunoblots were photodocumented for image analysis in the ImageJ free software. The expression of p21 and MCPIP1 proteins were normalized with β-Actin for subsequent analysis.

### Reanalysis of publicly available microarray datasets

We searched ZC3H12A/MCPIP1 and CDKN1A/p21 gene expression data from published studies that evaluated transcriptome in different cell populations of HIV-infected individuals through the Gene Expression Browser (GXB)—http://hiv.gxbsidra.org/dm3/geneBrowser/list. Raw expression profile data, with special focus on the genes of interest, ZC3H12A and CDKN1A, were obtained from six studies [33–38] through the Gene Expression Omnibus (GEO, http://www.ncbi.nlm.nih.gov/geo). In studies that used the Illumina microarray platform, the ZC3H12A gene was represented by one probe (ILMN_1672295) while the CDKN1A gene was represented by two probes (ILMN_1784602 and ILMN_1787212). In the study that used the Affymetrix Human Clariom D Assay, the ZC3H12A (TC0100007832.hg.1) and CDKN1A (TC0600007847.hg.1) genes were both represented by one probe. Two datasets (GSE18233 and GSE23879) reported expression profiles in CD4^+^ T cells from EC, ART-suppressed and HIV-negative subjects (Additional file 1: Tables S3 and S4). The third dataset (GSE28128) reported expression profiles in CD4^+^ T and CD8^+^ T cells from EC, NC-LTNP and NC-TP subjects (Additional file 1: Table S5). The fourth dataset (GSE129872/GSE128296) reported expression profiles in HIV-specific CD4^+^ T cells from EC, NC-TP and ART-suppressed subjects (Additional file 1: Table S6). The fifth dataset (GSE52900) reported expression profiles in monocytes from ART-suppressed, ART-non-suppressed and HIV-negative subjects (Additional file 1: Table S7). The sixth dataset (GSE18464) reported expression profiles in monocytes from High and Low -viral loads and HIV-negative subjects (Additional file 1: Table S8). Raw expression profile data from these six studies were normalized and transformed to log2 using the limma R-package [75], and correlations were performed for both genes.

### T cell and monocyte activation analyses

We used data of T cell and monocyte activation obtained in a previous study conducted by our group including these patients [73], in which plasma levels of soluble CD14 (sCD14) were determined by ELISA-sCD14 Quantikine assay (R&D Systems Minneapolis, MN) according to the manufacturer’s protocol and surface expression of combined HLA-DR and CD38 on CD4^+^ and CD8^+^ T cells was analyzed by flow cytometry.

### Data analyses

The comparisons of mean log-fold changes (log-FC) in mRNA abundance were performed by either t-tests or one-way ANOVA nonparametric permutation tests (B = 1000 permutations), followed by pair-wise comparisons with Holm-Bonferroni adjustment [76], for two or more groups respectively. The differential expression analysis from studies containing public data from EC individuals was performed with the limma R-package [75]. LogFC and FDR values were obtained for both genes (two/three probes) for all comparisons. Spearman coefficient was used for correlation analyses. A first-order log-Normal multiple regression analysis was fitted to model p21 gene expression as a function of MCPIP1 gene expression, CD4^+^ T cell activation (HLA-DR^+^CD38^+^), and HIC groups (EC and VC). The threshold for statistical significance was set to P < 0.05. Data were analyzed with R software (version 3.5.2) [77].

## Supplementary information

**Additional file 1.** Extended Materials with information about the cohorts of HIV-infected and uninfected subjects (Tables S2–S8) used in this study, and supplementary results (Figures S1–S11 and Table S1) regarding ZC3H12A/MCPIP1 and CDKN1A/p21 mRNA and protein expression levels, classical restriction factors mRNA expression and immune activation.

## Data Availability

The datasets during and/or analyzed during the current study available from the corresponding author on reasonable request.

## Abbreviations

HIV: Human immunodeficiency virus
HIC: HIV controllers
EC: Elite controllers
VC: Viremic controllers
PBMC: Peripheral blood mononuclear cells
VL: Viral loads
LOD: Limit of detection
RF: Restriction factors
APOBEC3G: Apolipoprotein B mRNA-Editing enzyme, Catalytic polypeptide-like
BST2: Bone Stromal Tumor protein 2
SAMHD1: Sterile Alpha Motif domain and HD domain-containing protein 1 (SAMHD1)
Mx: Myxovirus resistance protein
IFITM: Interferon-inducible transmembrane family proteins
SLFN11: Schlafen 11
ART: Antiretroviral
NEG: Healthy HIV-1-uninfected individuals
CDKN1A: Cyclin-dependent kinase (CDK) inhibitor p21
MCPIP1: Monocyte chemotactic protein–induced protein 1
MALT1: Mucosa-associated lymphoid-tissue lymphoma-translocation 1
Caki-1 cells: Human renal carcinoma cell line
KLF4: Kruppel-like factor 4
RIN: RNA integrity number
NC-TP: Untreated non-controllers typical progressors
NC-LTNP: Untreated non-controllers long-term nonprogressors
