# Supplementary material for: Increased expression of CDKN1A/p21 in HIV-1 controllers is correlated with upregulation of ZC3H12A/MCPIP1
Source: Retrovirology. 2020 Jul 2;17:18. doi: 10.1186/s12977-020-00522-4 (PMC7333275; doi:10.1186/s12977-020-00522-4)
Supplement: Supplementary file 1 — Additional file 1. Extended Materials with information about the cohorts of HIV-infected and uninfected subjects (Tables S2–S8) used in this study, and supplementary results (Figures S1–S11 and Table S1) regarding ZC3H12A/MCPIP1 and CDKN1A/p21 mRNA and protein expression levels, classical restriction factors mRNA expression and immune activation. [file 12977_2020_522_MOESM1_ESM.pdf]

Additional File

Increased expression of CDKN1A/p21 in HIV-1 controllers is correlated with upregulation of ZC3H12A/MCPIP1

Suwellen S. D. de Azevedo[1*], Marcelo Ribeiro-Alves[2], Fernanda H. Côrtes[1], Edson Delatorre[3], Lucia Spangenberg[4,5], Hugo Naya[5,6], Leonardo N. Seito[7], Brenda Hoagland[2], Beatriz Grinsztejn[2], Valdilea G. Veloso[2], Mariza G. Morgado[1], Thiago Moreno L. Souza[8,9], and Gonzalo Bello[1]

1 Laboratório de AIDS & Imunologia Molecular. Instituto Oswaldo Cruz – IOC, FIOCRUZ. Rio de Janeiro, Brazil.

2 Laboratório de Pesquisa Clínica em DST-AIDS. Instituto Nacional de Infectologia Evandro Chagas - INI, FIOCRUZ. Rio de Janeiro, Brazil.

3 Departamento de Biologia, Centro de Ciências Exatas, Naturais e da Saúde, Universidade Federal do Espírito Santo. Alegre, Brazil.

4 Unidad de Bioinformática, Institut Pasteur Montevideo. Montevideo, Uruguay.

5 Departamento de Informática y Ciencias de la Computación, Facultad de Ingeniería y Tecnologías, Universidad Católica del Uruguay, Montevideo, Uruguay.

6 Departamento de Producción Animal y Pasturas, Facultad de Agronomía, Universidad de la República, Montevideo, Uruguay.

7 Laboratório de Farmacologia Aplicada. Instituto de Tecnologia em Fármacos – Farmanguinhos, FIOCRUZ, Rio de Janeiro, Brazil.

8 National Institute for Science and Technology on Innovation on Diseases of Neglected Populations (INCT/IDPN), Center for Technological Development in Health – CDTS, FIOCRUZ. Rio de Janeiro, Brazil.

9 Laboratório de Imunofarmacologia. Instituto Oswaldo Cruz – IOC, FIOCRUZ. Rio de Janeiro, Brazil.

*** Corresponding author:** Suwellen de Azevedo. Lab. de AIDS & Imunologia Molecular. Instituto Oswaldo Cruz –FIOCRUZ. Av. Brasil 4365, 21045-900 Rio de Janeiro, RJ, Brasil. E-mail: suwellen@ioc.fiocruz.br/suwellendias@gmail.com

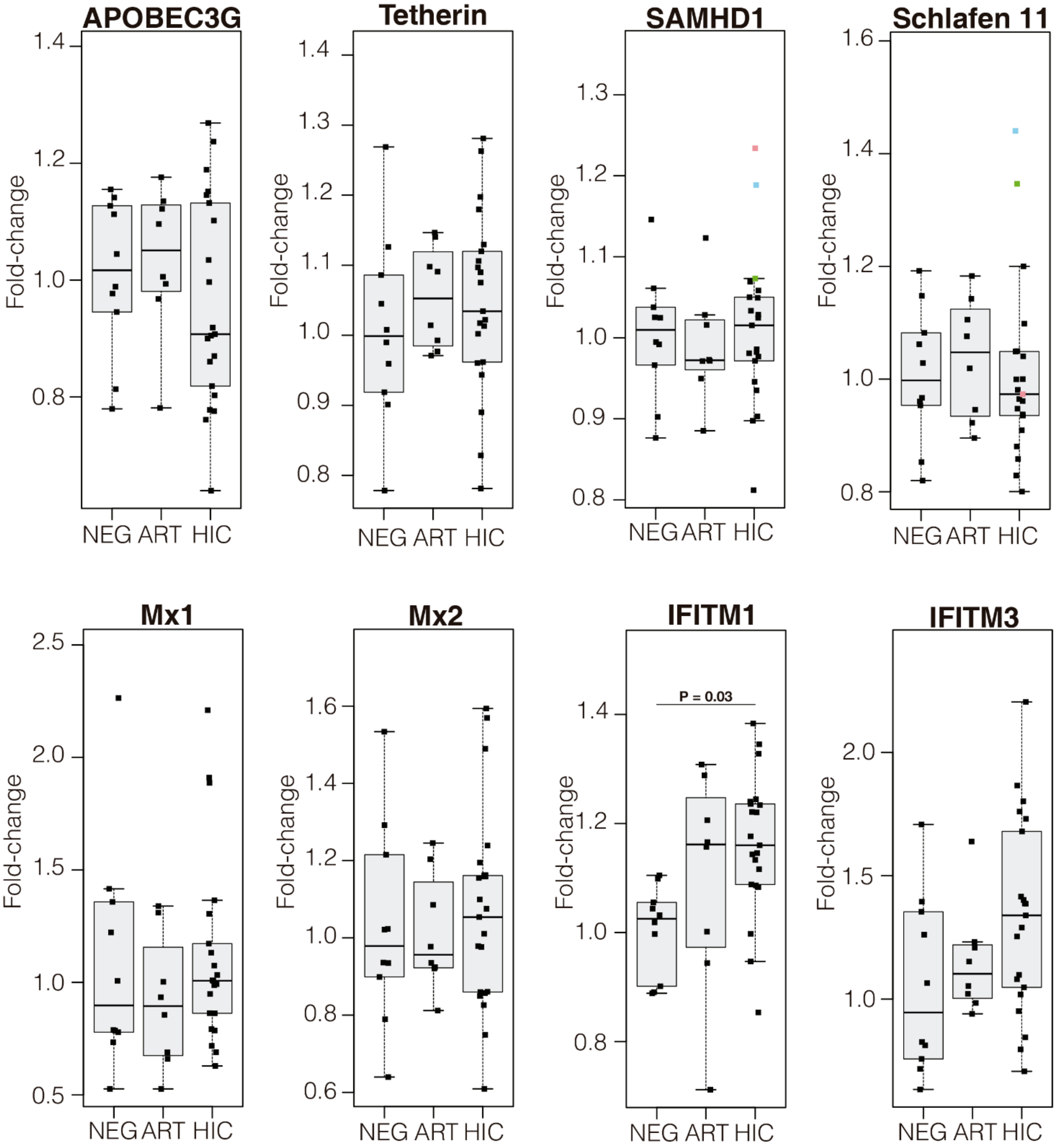

Figure S1. Boxplots represent the interquartile and sample median (central solid black line) of the relative changes (fold-change values relative to the mean of HIV-1-uninfected (NEG) subjects) of different restriction factor comparing NEG and ART-suppressed subjects (ART) with HIV controllers (HIC). The RF's names used in the analysis are indicated above each graph. HIC exhibited statistically significant differences (P-values < 0.05) with respect to NEG group only for IFITM1. The colored squares (one per individual) in SAMHD1 and Schlafen 11 from HIC represent individuals with mRNA levels well above the normal range in one or both RF.

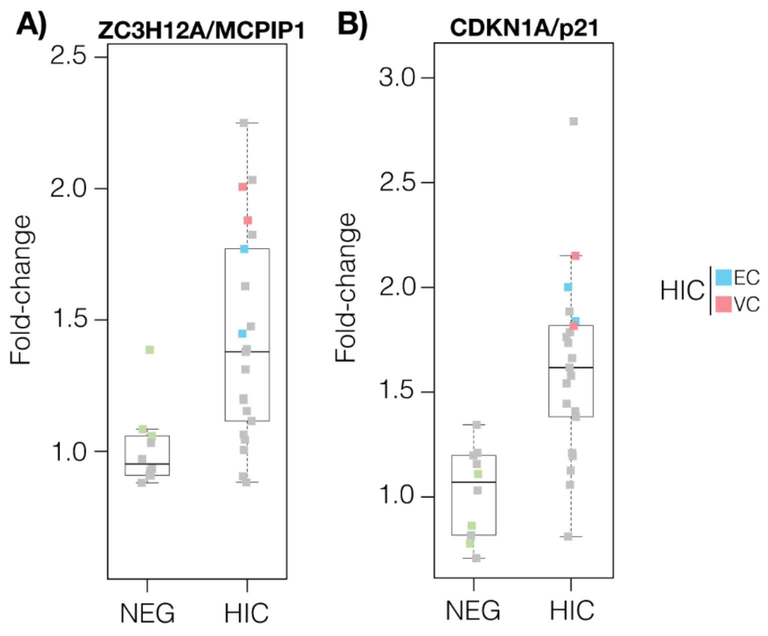

Figure S2. Samples chosen for analysis of MCPIP1 and p21 protein expression. Boxplots represent the interquartile and sample median (central solid black line) of the relative changes (fold-change values relative to the mean of HIV-1-uninfected (NEG) subjects) of ZC3H12A/MCPIP1 (A) and CDKN1A/p21 (B) expression. The colored squares represent the samples chosen for the western blot assay in the negative and HIC groups.

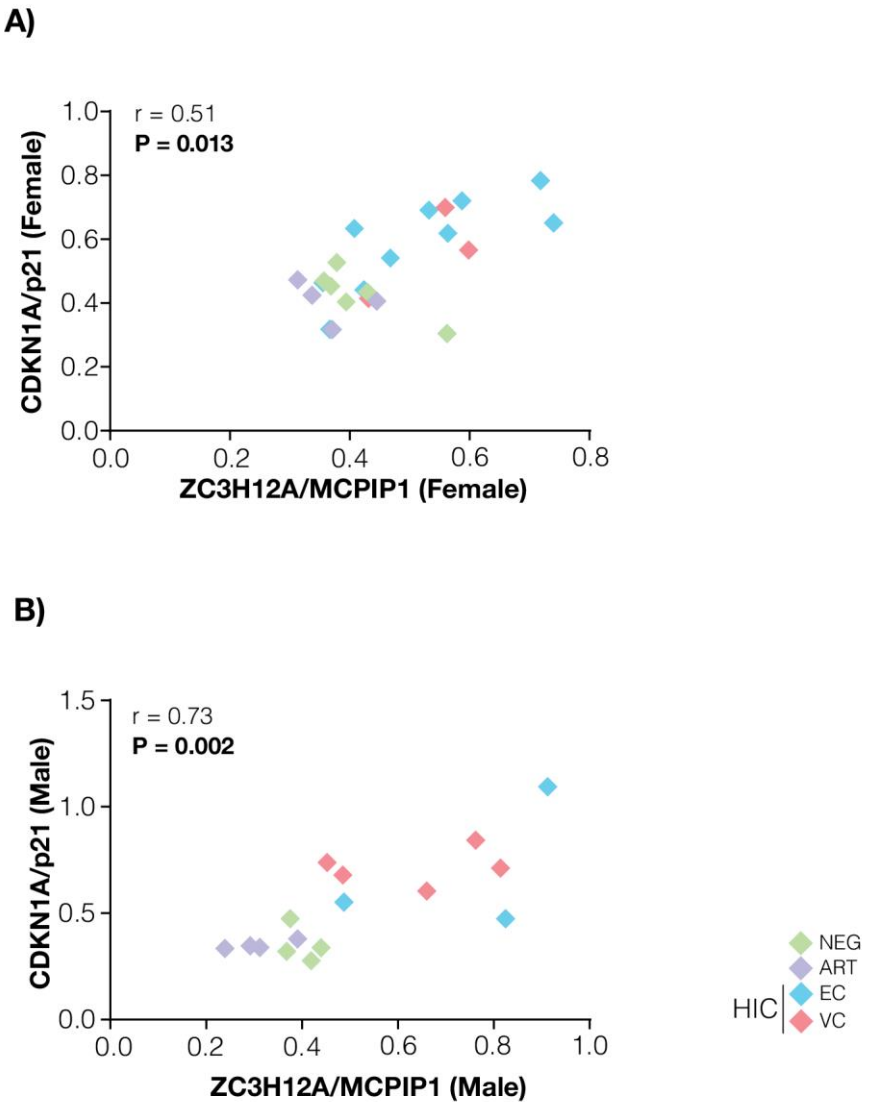

Figure S3. ZC3H12A/MCPIP1 and CDKN1A/p21 mRNA levels in PBMC are positively correlated, regardless of sex. The MCPIP1 and p21 normalized expression correlations were calculated considering all groups. The points' colors indicate the patient group, accordingly to the legend. Correlation coefficients (Spearman's ρ) are shown in the upper left corner of each graph. P-values < 0.05 were considered statistically significant.

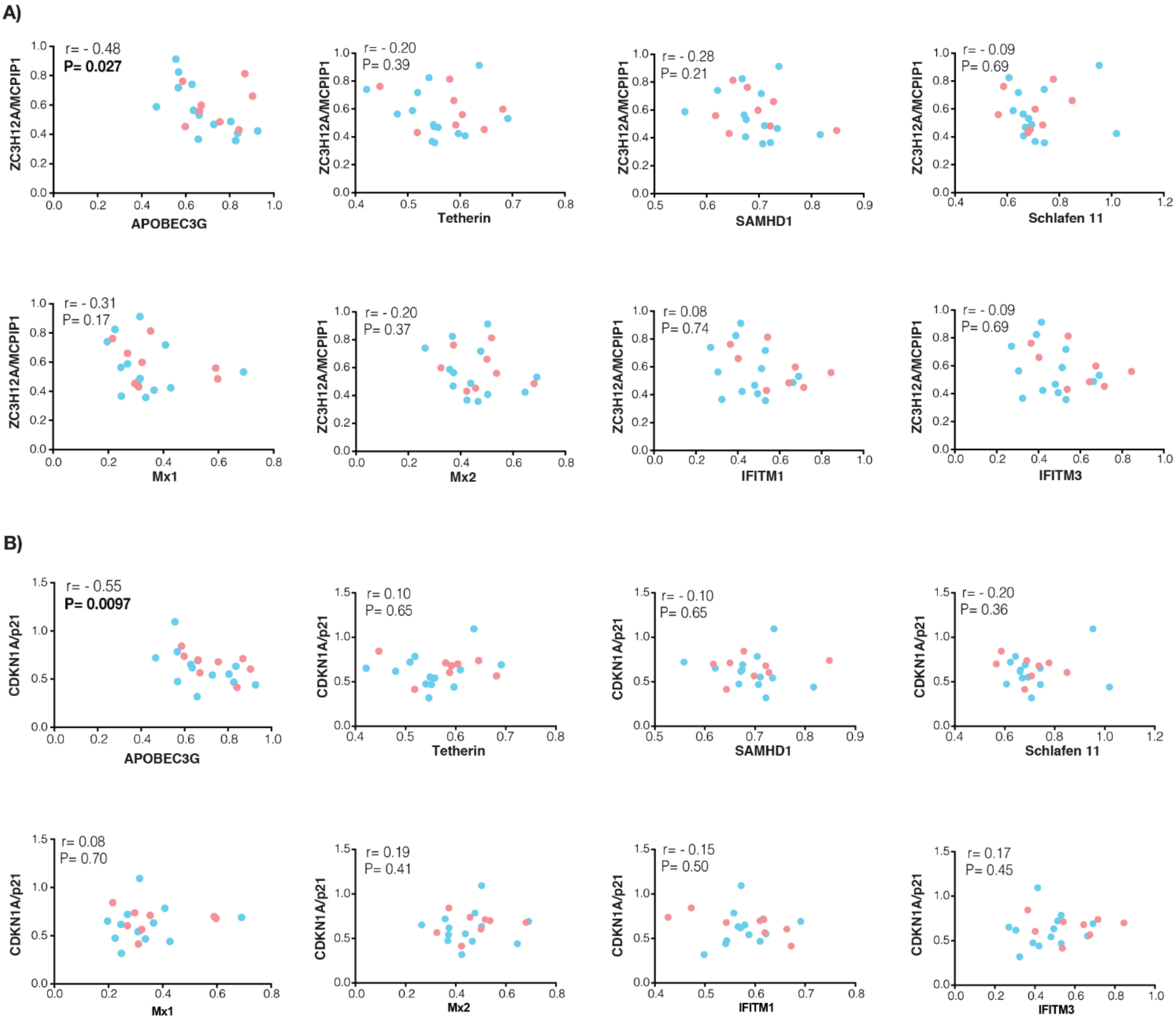

Figure S4. Correlations between normalized expression levels of ZC3H12A/MCPIP1 (A) and CDKN1A/p21 (B) with several anti-HIV-1 restriction factors (RF) in HIC. Blue points represent values form elite controllers while the red ones represent values from viremic controllers. The RF's names used in the correlation are indicated on the x-axis and the corresponding correlation coefficient (Spearman's rho) are shown in each graph.

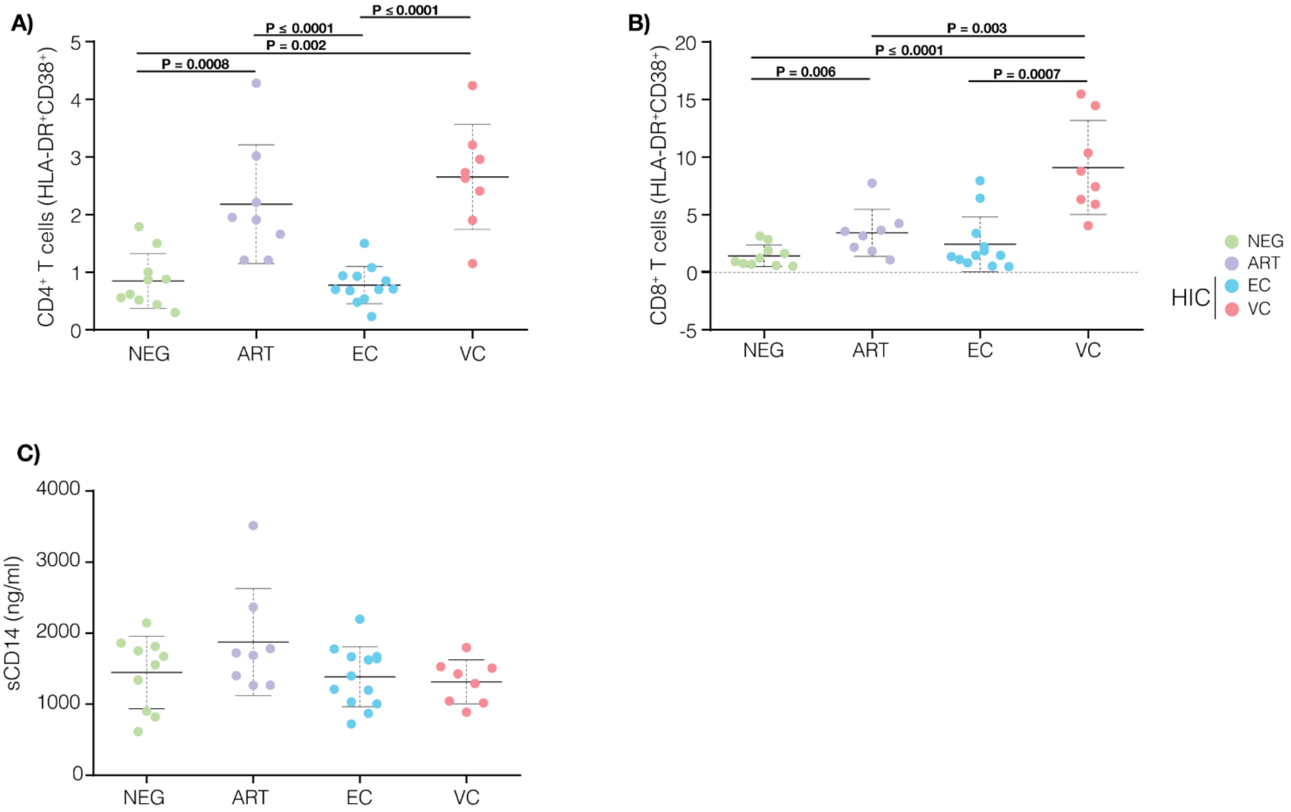

Figure S5. The mean of activated CD4$^+$ T cells (HLA-DR$^+$CD38$^+$) counts (A), activated CD8$^+$ T cells (HLA-DR$^+$CD38$^+$) counts (B), and soluble CD14 (sCD14) in plasma (C) were compared for each group. The color of each dot represents the group as indicated in the legend at right. P-values < 0.05 were considered statistically significant.

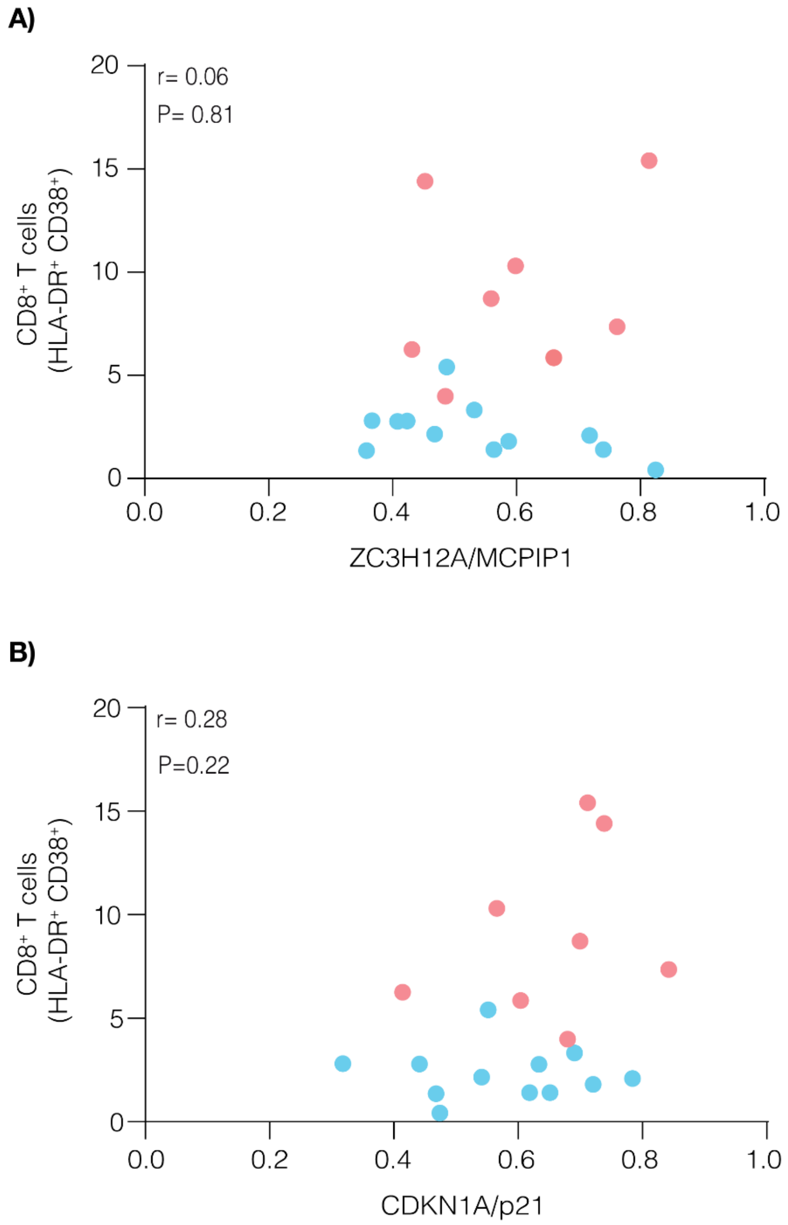

Figure S6. ZC3H12A/MCPIP1 and CDKN1A/p21 are not correlated with $CD8^{+}$ T cell in HIC individuals. Correlations were made evaluating the relationship between activated $CD8^{+}$ T cells levels with the normalized expression of ZC3H12A/MCPIP1 (A) and CDKN1A/p21 (B). Blue points represent values form elite controllers while the red ones represent values from viremic controllers. The correlation coefficient (Spearman's rho) are shown in the left corner.

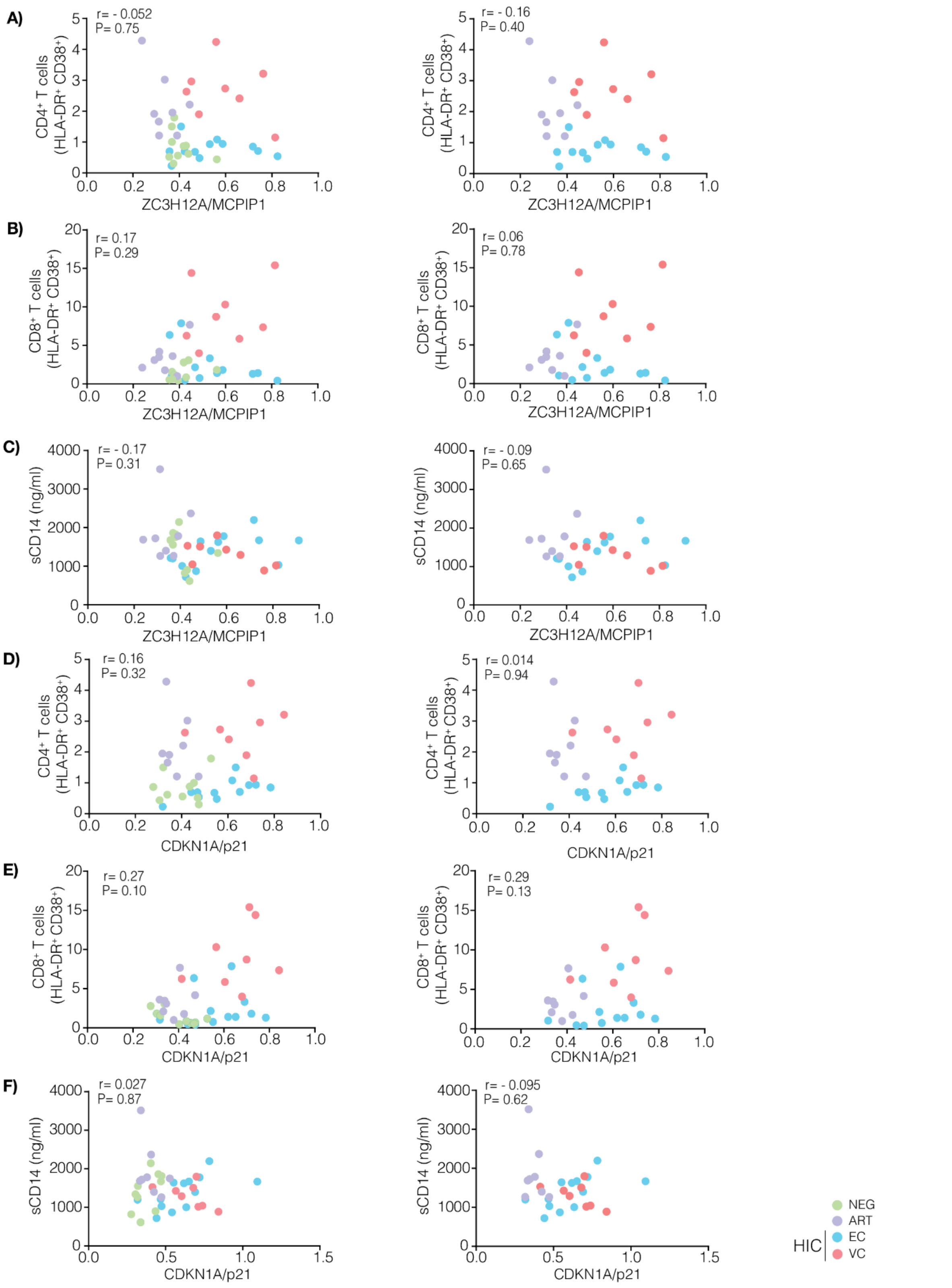

Figure S7. ZC3H12A/MCPIP1 and CDKN1A/p21 are not correlated with $CD4^+/CD8^+$ T cell, and monocyte activation in all groups and in HIV-1 infected individuals. Correlations were made evaluating the relationship between activated $CD4^+$, $CD8^+$ T cells or sCD14 levels with the normalized expression of ZC3H12A/MCPIP1 (A, B, and C, respectively) and CDKN1A/p21 (D, E and F, respectively) for different combinations of groups. The points' colors present in each graph indicate the groups present according to the legend. The correlation coefficient (Spearman's rho) are shown in the left corner.

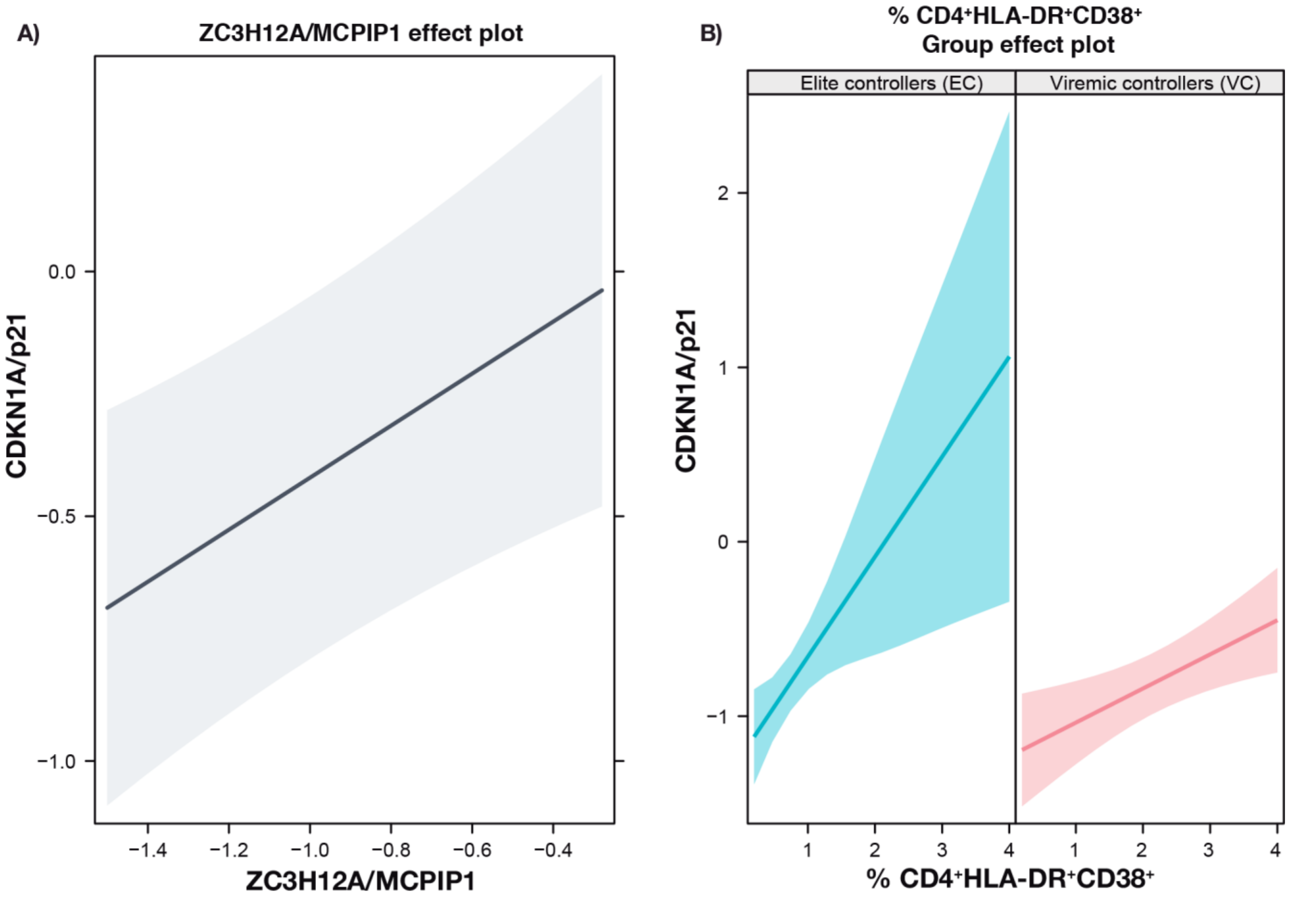

Figure S8. ZC3H12A/MCPIP1 and the frequency of CD4$^+$HLA-DR$^+$CD38$^+$ T cells upregulate CDKN1A/p21 mRNA levels in PBMC from HIC. Effects plots demonstrating (A) the upregulation of ZC3H12A/MCPIP1 is positively associated with the increase of the expression of CDKN1A/p21 in PBMC from HIC; while in (B) the frequency of CD4$^+$HLA-DR$^+$CD38$^+$ T cells is positively associated with the increase of the expression of CDKN1A/p21 in PBMC from both elite (EC) and viremic (VC) controllers, and this increase was of the p21 expression was down-regulated by the increase of CD4$^+$HLA-DR$^+$CD38$^+$ T cells in VC when compared to EC individuals. P-values < 0.05 were considered statistically significant.

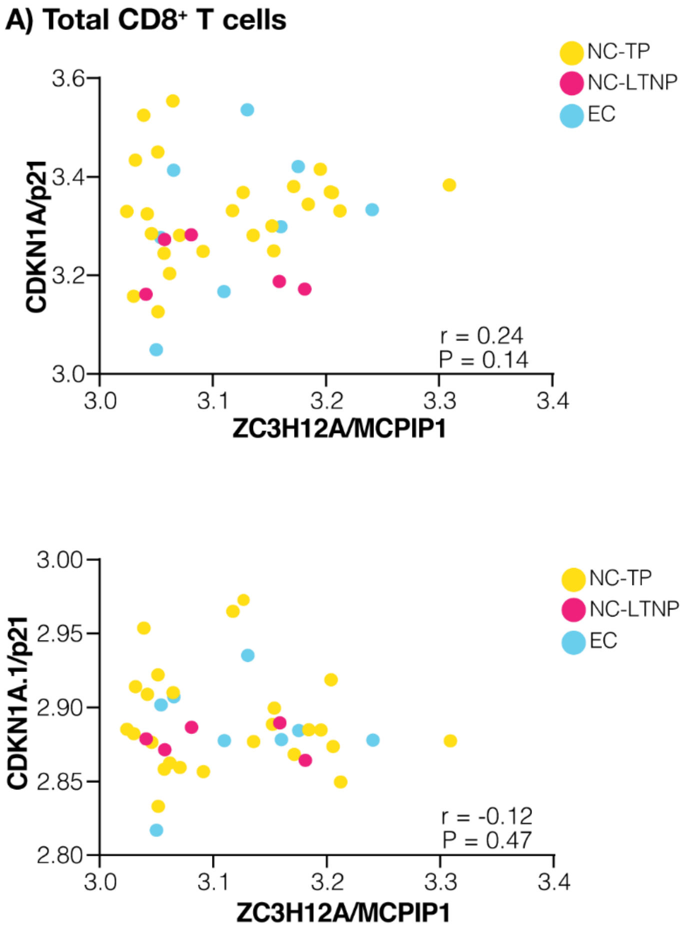

Figure S9: ZC3H12A/MCPIP1 and isoform of CDKN1A.1/p21 (probe ILMN_1787212) are not correlated with CD8^{+} T cell in all groups. Correlations were made using the expression data from previous study (GSE28128). The points' colors present in each graph indicate the groups present according to the legend. The correlation coefficient (Spearman's rho) are shown in the bottom right corner.

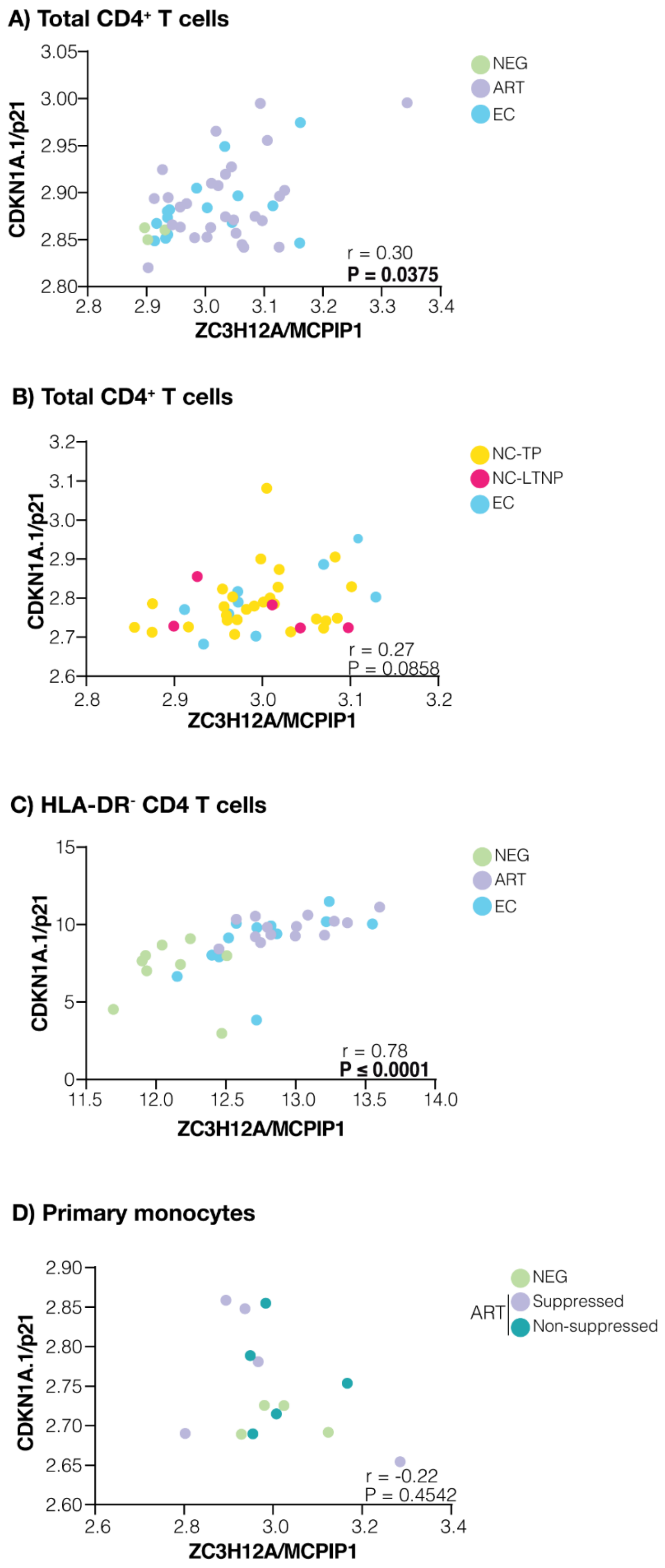

Figure S10: Correlations between ZC3H12A/MCPIP1 and isoform CDKN1A.1/p21 (ILMN_1787212) using the expression data from previous studies in A) and B) Total CD4$^+$ T cells (GSE18233, GSE28128, respectively), C) HLA-DR$^-$ CD4 T cells (GSE23879), and D) Primary monocytes (GSE52900). The points' colors present in each graph indicate the groups present according to the legend. The correlation coefficient (Spearman's rho) are shown in the bottom right corner.

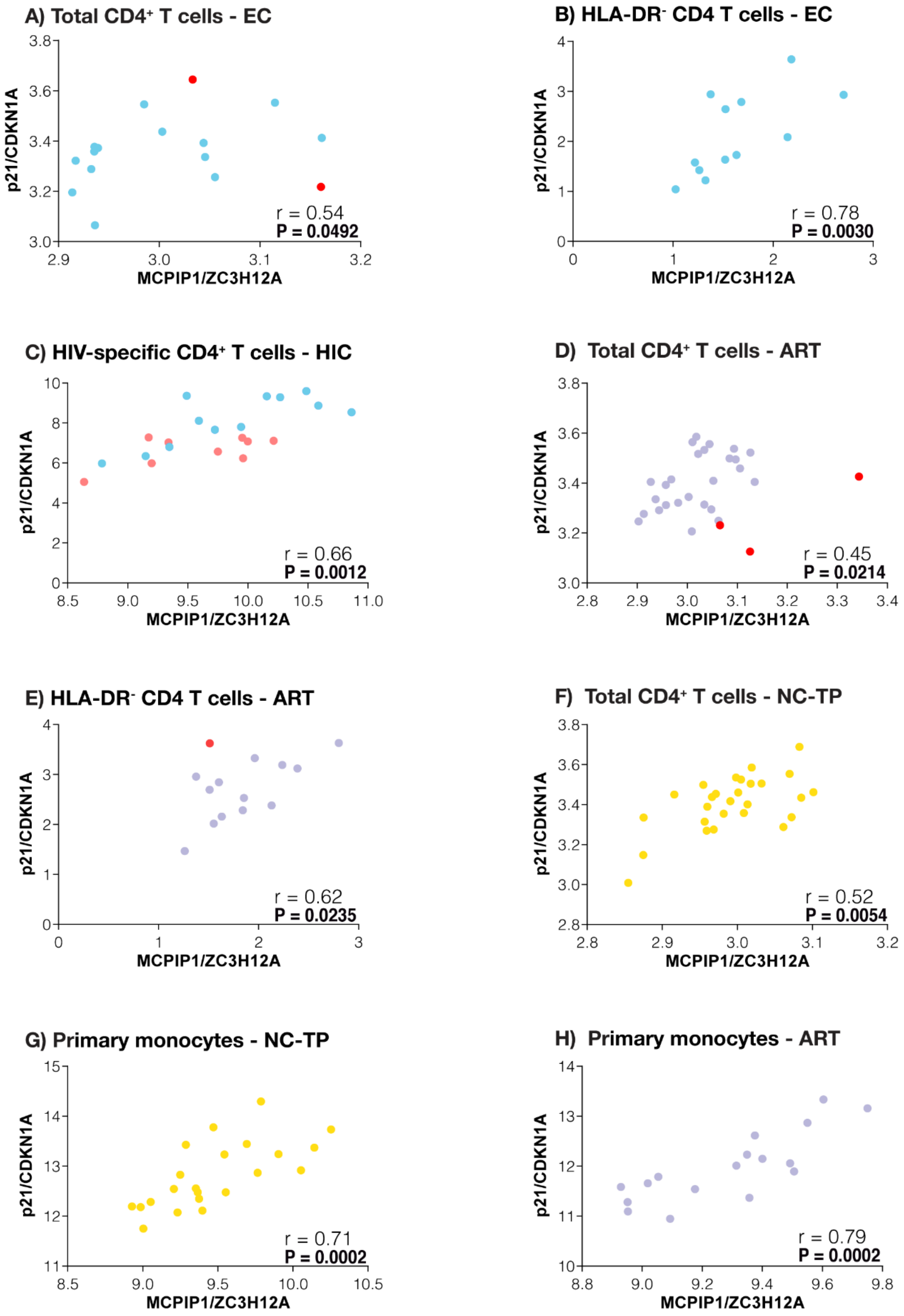

Figure S11: Correlations between ZC3H12A/MCPIP1 and CDKN1A/p21 using the expression data from previous studies in A) and D) Total CD4$^+$ T cells (GSE18233), B) and E) HLA-DR$^-$ CD4 T cells (GSE23879), C) HIV-specific CD4$^+$ T cells (GSE129872/128296), F) Total CD4$^+$ T cells (GSE28128) and G) and H) Primary monocytes (GSE18464). The points were colored following the color pattern of Figure 5. Outliers are showed in red. The correlation coefficient (Spearman's rho) are shown in the bottom right corner.

Table S1. Distribution of individuals in different groups according to sex.

Group	n (%)	Sex (n [%])		*P- value**
		**Female**	**Male**	
NEG	10 (25.6)	6 (15.4)	4 (10.3)	0.9083
ART	8 (20.5)	4 (10.3)	4 (10.3)	
HIC	21 (53.8)	13 (33.3)	8 (20.5)	
NEG	10 (25.6)	6 (15.4)	4 (10.3)	0.3273
ART	8 (20.5)	4 (10.3)	4 (10.3)	
EC	13 (33.3)	10 (25.6)	3 (7.7)	
VC	8 (20.5)	3 (7.7)	5 (12.8)	

* Statistical analyses were performed using the Fisher exact test.

Table S2. Differential expression analysis from ZC3H12A and CDKN1A genes in different cell types.

GEO accession number	Probe ID	Gene symbol	Cell Type	Groups	logFC	p-value
GSE23879 (Vigneault *et al.*2011)	ILMN_1672295	ZC3H12A	HLA-DR^{-} CD4 T cells	EC vs ART	0.19	0.186
	ILMN_1784602	CDKN1A	HLA-DR^{-} CD4 T cells		0.41	0.029
	ILMN_1787212	CDKN1A.1	HLA-DR^{-} CD4 T cells		0.92	0.104
	ILMN_1672295	ZC3H12A	HLA-DR^{-} CD4 T cells	NEG vs EC	-0.65	0.000
	ILMN_1784602	CDKN1A	HLA-DR^{-} CD4 T cells		-0.99	0.000
	ILMN_1787212	CDKN1A.1	HLA-DR^{-} CD4 T cells		-1.62	0.049
	ILMN_1672295	ZC3H12A	HLA-DR^{-} CD4 T cells	ART vs NEG	0.84	0.000
	ILMN_1784602	CDKN1A	HLA-DR^{-} CD4 T cells		1.41	0.000
	ILMN_1787212	CDKN1A.1	HLA-DR^{-} CD4 T cells		2.54	0.000
GSE18233 (Rotger *et al.*2010)	ILMN_1672295	ZC3H12A	Total CD4^{+} T cells	EC vs ART	-0.13	0.322
	ILMN_1784602	CDKN1A	Total CD4^{+} T cells		-0.18	0.527
	ILMN_1787212	CDKN1A.1	Total CD4^{+} T cells		-0.05	0.525
	ILMN_1672295	ZC3H12A	Total CD4^{+} T cells	NEG vs EC	-0.58	0.003
	ILMN_1784602	CDKN1A	Total CD4^{+} T cells		-1.11	0.006
	ILMN_1787212	CDKN1A.1	Total CD4^{+} T cells		-0.13	0.047
	ILMN_1672295	ZC3H12A	Total CD4^{+} T cells	ART vs NEG	0.71	0.000
	ILMN_1784602	CDKN1A	Total CD4^{+} T cells		1.29	0.000
	ILMN_1787212	CDKN1A.1	Total CD4^{+} T cells		0.18	0.036
GSE28128 (Rotger *et al.*2011)	ILMN_1672295	ZC3H12A	Total CD4^{+} T cells	EC vs NC-TP	-0.07	0.591
	ILMN_1784602	CDKN1A	Total CD4^{+} T cells		0.19	0.604
	ILMN_1787212	CDKN1A.1	Total CD4^{+} T cells		-0.03	0.845
	ILMN_1672295	ZC3H12A	Total CD4^{+} T cells	EC vs NC-LTNP	-0.06	0.804
	ILMN_1784602	CDKN1A	Total CD4^{+} T cells		0.11	0.838
	ILMN_1787212	CDKN1A.1	Total CD4^{+} T cells		-0.16	0.398
	ILMN_1672295	ZC3H12A	Total CD4^{+} T cells	NC-LTNP vs NC-TP	-0.01	0.912
	ILMN_1784602	CDKN1A	Total CD4^{+} T cells		0.08	0.856
	ILMN_1787212	CDKN1A.1	Total CD4^{+} T cells		0.13	0.465
GSE129872/128296 (Morou *et al.*2019)	TC0100007832.hg.1	ZC3H12A	HIV-specific CD4^{+} T cells	NC-TP vs EC	-0.66	0.007
	TC0600007847.hg.1	CDKN1A	HIV-specific CD4^{+} T cells		-2.13	0.000
	TC0100007832.hg.1	ZC3H12A	HIV-specific CD4^{+} T cells	ART vs EC	-0.60	0.017
	TC0600007847.hg.1	CDKN1A	HIV-specific CD4^{+} T cells		-1.47	0.002
	TC0100007832.hg.1	ZC3H12A	HIV-specific CD4^{+} T cells	EC vs VC	-0.28	0.249
	TC0600007847.hg.1	CDKN1A	HIV-specific CD4^{+} T cells		-1.52	0.001
	TC0100007832.hg.1	ZC3H12A	HIV-specific CD4^{+} T cells	HIC vs NC-TP	0.53	0.011
	TC0600007847.hg.1	CDKN1A	HIV-specific CD4^{+} T cells		1.48	0.000
	TC0100007832.hg.1	ZC3H12A	HIV-specific CD4^{+} T cells	NC-TP vs VC	-0.37	0.092
	TC0600007847.hg.1	CDKN1A	HIV-specific CD4^{+} T cells		-0.61	0.071

EC: Elite controllers; ART: Successfully treated-progressor; NEG: HIV negative; NC-TP: Untreated non-controllers typical progressors; NC-LTNP: Untreated long-term nonprogressors; HIC: EC+VC;

Table S3. List of samples from Rotger et al. 2010 used in this study.

GEO accession number	Barcode	Sample Title	Group	Cell Type
GSE18233 (Rotger *et al*.2010)	GSM455600	Untreated 3011	Elite controller	Total CD4$^+$ T cells
	GSM455601	Untreated 3013	Elite controller	Total CD4$^+$ T cells
	GSM455602	Untreated 3130	Elite controller	Total CD4$^+$ T cells
	GSM455604	Untreated 3336	Elite controller	Total CD4$^+$ T cells
	GSM455609	Untreated 3595	Elite controller	Total CD4$^+$ T cells
	GSM455611	Untreated 3713	Elite controller	Total CD4$^+$ T cells
	GSM455613	Untreated 3929	Elite controller	Total CD4$^+$ T cells
	GSM455615	Untreated 5076	Elite controller	Total CD4$^+$ T cells
	GSM455737	Untreated 5539	Elite controller	Total CD4$^+$ T cells
	GSM455741	Untreated 5544	Elite controller	Total CD4$^+$ T cells
	GSM455616	Untreated 5627	Elite controller	Total CD4$^+$ T cells
	GSM455622	Untreated 5646	Elite controller	Total CD4$^+$ T cells
	GSM455623	Untreated 5647	Elite controller	Total CD4$^+$ T cells
	GSM455644	Untreated 5681	Elite controller	Total CD4$^+$ T cells
	GSM455646	Untreated 5683	Elite controller	Total CD4$^+$ T cells
	GSM455650	Untreated 5687	Elite controller	Total CD4$^+$ T cells
	GSM455596	Succesfully treated 3073	Progressor (Succesfully treated)	Total CD4$^+$ T cells
	GSM455659	Succesfully treated 3085	Progressor (Succesfully treated)	Total CD4$^+$ T cells
	GSM455661	Succesfully treated 3149	Progressor (Succesfully treated)	Total CD4$^+$ T cells
	GSM455662	Succesfully treated 3152	Progressor (Succesfully treated)	Total CD4$^+$ T cells
	GSM455672	Succesfully treated 3307	Progressor (Succesfully treated)	Total CD4$^+$ T cells
	GSM455674	Succesfully treated 3477	Progressor (Succesfully treated)	Total CD4$^+$ T cells
	GSM455582	Succesfully treated 3487	Progressor (Succesfully treated)	Total CD4$^+$ T cells
	GSM455583	Succesfully treated 3862	Progressor (Succesfully treated)	Total CD4$^+$ T cells
	GSM455683	Succesfully treated 3937	Progressor (Succesfully treated)	Total CD4$^+$ T cells
	GSM455687	Succesfully treated 5136	Progressor (Succesfully treated)	Total CD4$^+$ T cells
	GSM455688	Succesfully treated 5140	Progressor (Succesfully treated)	Total CD4$^+$ T cells
	GSM455696	Succesfully treated 5483	Progressor (Succesfully treated)	Total CD4$^+$ T cells
	GSM455698	Succesfully treated 5490	Progressor (Succesfully treated)	Total CD4$^+$ T cells
	GSM455703	Succesfully treated 5496	Progressor (Succesfully treated)	Total CD4$^+$ T cells
	GSM455708	Succesfully treated 5499	Progressor (Succesfully treated)	Total CD4$^+$ T cells
	GSM455725	Succesfully treated 5520	Progressor (Succesfully treated)	Total CD4$^+$ T cells
	GSM455594	Succesfully treated 5567	Progressor (Succesfully treated)	Total CD4$^+$ T cells
	GSM455766	Succesfully treated 5619	Progressor (Succesfully treated)	Total CD4$^+$ T cells
	GSM455768	Succesfully treated 5622	Progressor (Succesfully treated)	Total CD4$^+$ T cells
	GSM455772	Succesfully treated 5628	Progressor (Succesfully treated)	Total CD4$^+$ T cells
	GSM455617	Succesfully treated 5633	Progressor (Succesfully treated)	Total CD4$^+$ T cells
	GSM455618	Succesfully treated 5637	Progressor (Succesfully treated)	Total CD4$^+$ T cells
	GSM455628	Succesfully treated 5652	Progressor (Succesfully treated)	Total CD4$^+$ T cells
	GSM455629	Succesfully treated 5659	Progressor (Succesfully treated)	Total CD4$^+$ T cells
	GSM455637	Succesfully treated 5672	Progressor (Succesfully treated)	Total CD4$^+$ T cells
	GSM455648	Succesfully treated 5685	Progressor (Succesfully treated)	Total CD4$^+$ T cells
	GSM455653	Succesfully treated 5699	Progressor (Succesfully treated)	Total CD4$^+$ T cells
	GSM455656	Succesfully treated 5703	Progressor (Succesfully treated)	Total CD4$^+$ T cells
	GSM455657	Succesfully treated 5704	Progressor (Succesfully treated)	Total CD4$^+$ T cells
	GSM455777	Uninfected_rep1 pull 2 ATE 105	HIV negative	Total CD4$^+$ T cells
	GSM455779	Uninfected_rep1 pull 3_ATE 048	HIV negative	Total CD4$^+$ T cells
	GSM455774	Uninfected_rep2 pull 1_ATE 078	HIV negative	Total CD4$^+$ T cells
	GSM455778	Uninfected_rep2 pull 2_ATE 108	HIV negative	Total CD4$^+$ T cells
	GSM455780	Uninfected_rep2 pull 3_ATE 051	HIV negative	Total CD4$^+$ T cells
	GSM455775	Uninfected_rep3 pull 2_ATE 196	HIV negative	Total CD4$^+$ T cells
	GSM455781	Uninfected_rep3 pull 3 ATE 057	HIV negative	Total CD4$^+$ T cells
	GSM455776	Uninfected_rep1 pull 2 ATE 105	HIV negative	Total CD4$^+$ T cells

Table S4. List of samples from Vigneault et al. 2011 used in this study.

GEO accession number	Barcode	Sample Title	Group	Cell Type
GSE23879 (Vigneault *et al*.2011)	GSM588887	CD4 EC 01	Elite controller	HLA-DR^{-} CD4 T cells
	GSM588888	CD4 EC 02	Elite controller	HLA-DR^{-} CD4 T cells
	GSM588889	CD4 EC 03	Elite controller	HLA-DR^{-} CD4 T cells
	GSM588890	CD4 EC 04	Elite controller	HLA-DR^{-} CD4 T cells
	GSM588891	CD4 EC 05	Elite controller	HLA-DR^{-} CD4 T cells
	GSM588892	CD4 EC 06	Elite controller	HLA-DR^{-} CD4 T cells
	GSM588893	CD4 EC 07	Elite controller	HLA-DR^{-} CD4 T cells
	GSM588894	CD4 EC 08	Elite controller	HLA-DR^{-} CD4 T cells
	GSM588895	CD4 EC 09	Elite controller	HLA-DR^{-} CD4 T cells
	GSM588896	CD4 EC 10	Elite controller	HLA-DR^{-} CD4 T cells
	GSM588897	CD4 EC 11	Elite controller	HLA-DR^{-} CD4 T cells
	GSM588898	CD4 EC 12	Elite controller	HLA-DR^{-} CD4 T cells
	GSM588911	CD4 HIV+ 01	ART Treated	HLA-DR^{-} CD4 T cells
	GSM588912	CD4 HIV+ 02	ART Treated	HLA-DR^{-} CD4 T cells
	GSM588913	CD4 HIV+ 03	ART Treated	HLA-DR^{-} CD4 T cells
	GSM588914	CD4 HIV+ 04	ART Treated	HLA-DR^{-} CD4 T cells
	GSM588915	CD4 HIV+ 05	ART Treated	HLA-DR^{-} CD4 T cells
	GSM588916	CD4 HIV+ 06	ART Treated	HLA-DR^{-} CD4 T cells
	GSM588917	CD4 HIV+ 07	ART Treated	HLA-DR^{-} CD4 T cells
	GSM588918	CD4 HIV+ 08	ART Treated	HLA-DR^{-} CD4 T cells
	GSM588919	CD4 HIV+ 09	ART Treated	HLA-DR^{-} CD4 T cells
	GSM588920	CD4 HIV+ 10	ART Treated	HLA-DR^{-} CD4 T cells
	GSM588921	CD4 HIV+ 11	ART Treated	HLA-DR^{-} CD4 T cells
	GSM588922	CD4 HIV+ 12	ART Treated	HLA-DR^{-} CD4 T cells
	GSM588923	CD4 HIV+ 13_repl1	ART Treated	HLA-DR^{-} CD4 T cells
	GSM588924	CD4 HIV+ 13_repl2	ART Treated	HLA-DR^{-} CD4 T cells
	GSM588925	CD4 HIV+ 15	ART Treated	HLA-DR^{-} CD4 T cells
	GSM588899	CD4 HIV- 01	HIV negative	HLA-DR^{-} CD4 T cells
	GSM588900	CD4 HIV- 02	HIV negative	HLA-DR^{-} CD4 T cells
	GSM588901	CD4 HIV- 03_repl1	HIV negative	HLA-DR^{-} CD4 T cells
	GSM588902	CD4 HIV- 03_repl2	HIV negative	HLA-DR^{-} CD4 T cells
	GSM588903	CD4 HIV- 04	HIV negative	HLA-DR^{-} CD4 T cells
	GSM588904	CD4 HIV- 05	HIV negative	HLA-DR^{-} CD4 T cells
	GSM588905	CD4 HIV- 06	HIV negative	HLA-DR^{-} CD4 T cells
	GSM588906	CD4 HIV- 07_repl1	HIV negative	HLA-DR^{-} CD4 T cells
	GSM588907	CD4 HIV- 07_repl2	HIV negative	HLA-DR^{-} CD4 T cells
	GSM588908	CD4 HIV- 08_repl1	HIV negative	HLA-DR^{-} CD4 T cells
	GSM588909	CD4 HIV- 08_repl2	HIV negative	HLA-DR^{-} CD4 T cells
	GSM588910	CD4 HIV- 09	HIV negative	HLA-DR^{-} CD4 T cells

Table S5. List of samples from Rotger et al. 2011 used in this study

GEO accession number	Barcode	Sample Title	Group	Cell Type
GSE28128 (Rotger *et al*.2011)	GSM696900	EC_3011	Elite controller	Total $CD4^+$ T cells
	GSM696901	EC_3336	Elite controller	Total $CD4^+$ T cells
	GSM696902	EC_3713	Elite controller	Total $CD4^+$ T cells
	GSM696903	EC_3984	Elite controller	Total $CD4^+$ T cells
	GSM696904	EC_5563	Elite controller	Total $CD4^+$ T cells
	GSM696905	EC_5627	Elite controller	Total $CD4^+$ T cells
	GSM696906	EC_5647	Elite controller	Total $CD4^+$ T cells
	GSM696907	EC_5681	Elite controller	Total $CD4^+$ T cells
	GSM696908	EC_5683	Elite controller	Total $CD4^+$ T cells
	GSM696909	*RP_3079	Rapid Progressor	Total $CD4^+$ T cells
	GSM696910	RP_3997	Rapid Progressor	Total $CD4^+$ T cells
	GSM696911	RP_5484	Rapid Progressor	Total $CD4^+$ T cells
	GSM696912	RP_5506	Rapid Progressor	Total $CD4^+$ T cells
	GSM696913	RP_5537	Rapid Progressor	Total $CD4^+$ T cells
	GSM696914	RP_5619	Rapid Progressor	Total $CD4^+$ T cells
	GSM696915	RP_5643	Rapid Progressor	Total $CD4^+$ T cells
	GSM696916	RP_5685	Rapid Progressor	Total $CD4^+$ T cells
	GSM696917	RP_7777	Rapid Progressor	Total $CD4^+$ T cells
	GSM696918	RP_7781	Rapid Progressor	Total $CD4^+$ T cells
	GSM696919	RP_7783	Rapid Progressor	Total $CD4^+$ T cells
	GSM696920	RP_HC13	Rapid Progressor	Total $CD4^+$ T cells
	GSM696921	RP_HC16	Rapid Progressor	Total $CD4^+$ T cells
	GSM696922	RP_IC330	Rapid Progressor	Total $CD4^+$ T cells
	GSM696923	RP_IC436	Rapid Progressor	Total $CD4^+$ T cells
	GSM696924	RP_IC443	Rapid Progressor	Total $CD4^+$ T cells
	GSM696925	RP_IC450	Rapid Progressor	Total $CD4^+$ T cells
	GSM696926	RP_IC458	Rapid Progressor	Total $CD4^+$ T cells
	GSM696927	RP_IC519	Rapid Progressor	Total $CD4^+$ T cells
	GSM696928	RP_IC527	Rapid Progressor	Total $CD4^+$ T cells
	GSM696929	RP_IC548	Rapid Progressor	Total $CD4^+$ T cells
	GSM696930	RP_IC590	Rapid Progressor	Total $CD4^+$ T cells
	GSM696931	RP_IC654	Rapid Progressor	Total $CD4^+$ T cells
	GSM696932	RP_IC678	Rapid Progressor	Total $CD4^+$ T cells
	GSM696933	RP_IC699	Rapid Progressor	Total $CD4^+$ T cells
	GSM696934	RP_IC720	Rapid Progressor	Total $CD4^+$ T cells
	GSM696935	RP_IC760	Rapid Progressor	Total $CD4^+$ T cells
	GSM696936	*VNP_6109	Viremic non-progressor	Total $CD4^+$ T cells
	GSM696937	VNP_6309	Viremic non-progressor	Total $CD4^+$ T cells
	GSM696938	VNP_7843	Viremic non-progressor	Total $CD4^+$ T cells
	GSM696939	VNP_7852	Viremic non-progressor	Total $CD4^+$ T cells
	GSM696940	VNP_IC820	Viremic non-progressor	Total $CD4^+$ T cells

*RP and VPN were renamed to NC-TP and NC-LTNP, respectively in our study.

Table S6. List of samples from Morou et al. 2019 used in this study

GEO accession number	Barcode	Sample Title	Group	Cell Type
GSE129872/128296 (Morou *et al.*2019)	GSM3723802	EC1	Elite controller	HIV-specific CD4^{+} T cells
	GSM3723803	EC2	Elite controller	HIV-specific CD4^{+} T cells
	GSM3723804	EC4	Elite controller	HIV-specific CD4^{+} T cells
	GSM3723805	EC5	Elite controller	HIV-specific CD4^{+} T cells
	GSM3723806	EC7	Elite controller	HIV-specific CD4^{+} T cells
	GSM3723807	EC8	Elite controller	HIV-specific CD4^{+} T cells
	GSM3723808	EC9	Elite controller	HIV-specific CD4^{+} T cells
	GSM3723809	EC10	Elite controller	HIV-specific CD4^{+} T cells
	GSM3723810	EC11	Elite controller	HIV-specific CD4^{+} T cells
	GSM3723811	EC12	Elite controller	HIV-specific CD4^{+} T cells
	GSM3723812	EC13	Elite controller	HIV-specific CD4^{+} T cells
	GSM3723813	EC15	Elite controller	HIV-specific CD4^{+} T cells
	GSM3723793	VC1	Viremic controller	HIV-specific CD4^{+} T cells
	GSM3723794	VC2	Viremic controller	HIV-specific CD4^{+} T cells
	GSM3723795	VC3	Viremic controller	HIV-specific CD4^{+} T cells
	GSM3723796	VC6	Viremic controller	HIV-specific CD4^{+} T cells
	GSM3723797	VC8	Viremic controller	HIV-specific CD4^{+} T cells
	GSM3723798	VC9	Viremic controller	HIV-specific CD4^{+} T cells
	GSM3723799	VC10	Viremic controller	HIV-specific CD4^{+} T cells
	GSM3723800	VC11	Viremic controller	HIV-specific CD4^{+} T cells
	GSM3723801	VC12	Viremic controller	HIV-specific CD4^{+} T cells
	GSM3723782	*CP1	Chronic progressor	HIV-specific CD4^{+} T cells
	GSM3723783	CP2	Chronic progressor	HIV-specific CD4^{+} T cells
	GSM3723784	CP3	Chronic progressor	HIV-specific CD4^{+} T cells
	GSM3723785	CP5	Chronic progressor	HIV-specific CD4^{+} T cells
	GSM3723786	CP6	Chronic progressor	HIV-specific CD4^{+} T cells
	GSM3723787	CP7	Chronic progressor	HIV-specific CD4^{+} T cells
	GSM3723788	CP8	Chronic progressor	HIV-specific CD4^{+} T cells
	GSM3723789	CP9	Chronic progressor	HIV-specific CD4^{+} T cells
	GSM3723790	CP10	Chronic progressor	HIV-specific CD4^{+} T cells
	GSM3723791	CP11	Chronic progressor	HIV-specific CD4^{+} T cells
	GSM3723792	CP12	Chronic progressor	HIV-specific CD4^{+} T cells
	GSM3670818	CP1_postART	Progressor (Succesfully treated)	HIV-specific CD4^{+} T cells
	GSM3670819	CP3_postART	Progressor (Succesfully treated)	HIV-specific CD4^{+} T cells
	GSM3670820	CP6_postART	Progressor (Succesfully treated)	HIV-specific CD4^{+} T cells
	GSM3670821	CP8_postART	Progressor (Succesfully treated)	HIV-specific CD4^{+} T cells
	GSM3670822	CP10_postART	Progressor (Succesfully treated)	HIV-specific CD4^{+} T cells
	GSM3670823	CP11_postART	Progressor (Succesfully treated)	HIV-specific CD4^{+} T cells
	GSM3670824	CP13_postART	Progressor (Succesfully treated)	HIV-specific CD4^{+} T cells
	GSM3670825	CP14_postART	Progressor (Succesfully treated)	HIV-specific CD4^{+} T cells

CP and CP_postART were renamed to NC-TP and ART, respectively in our study.

Table S7. List of samples from Wu et al. 2013 used in this study.

GEO accession number	Barcode	Sample Title	Group	Cell Type
GSE52900 (Wu *et al*.2013)	GSM1277694	BDL_biorep1*	ART_Below Detection Level	Primary monocytes
	GSM1277695	BDL_biorep2	ART_Below Detection Level	Primary monocytes
	GSM1277696	BDL_biorep3	ART_Below Detection Level	Primary monocytes
	GSM1277697	BDL_biorep4	ART_Below Detection Level	Primary monocytes
	GSM1277698	BDL_biorep5	ART_Below Detection Level	Primary monocytes
	GSM1277699	VIR_biorep1*	ART_ experiencing viremia	Primary monocytes
	GSM1277700	VIR_biorep2	ART_ experiencing viremia	Primary monocytes
	GSM1277701	VIR_biorep3	ART_ experiencing viremia	Primary monocytes
	GSM1277702	VIR_biorep4	ART_ experiencing viremia	Primary monocytes
	GSM1277703	VIR_biorep5	ART_ experiencing viremia	Primary monocytes
	GSM1277704	ctrl_biorep1	HIV Negative	Primary monocytes
	GSM1277705	ctrl_biorep2	HIV Negative	Primary monocytes
	GSM1277706	ctrl_biorep3	HIV Negative	Primary monocytes
	GSM1277707	ctrl_biorep4	HIV Negative	Primary monocytes

*BDL and VIR were renamed to ART-suppressed and ART non-suppressed, respectively in our study.

Table S8. List of samples from Rempel et al. 2010 used in this study.

GEO accession number	Barcode	Sample Title	Group	Cell Type
GSE18464 (Rempel *et al*.2010)	GSM361177	Monocyte_LVL*_002	Low-viral loads	Primary monocytes
	GSM361179	Monocyte_LVL_004	Low-viral loads	Primary monocytes
	GSM361180	Monocyte_LVL_005	Low-viral loads	Primary monocytes
	GSM361181	Monocyte_LVL_006	Low-viral loads	Primary monocytes
	GSM361182	Monocyte_LVL_007	Low-viral loads	Primary monocytes
	GSM361186	Monocyte_LVL_013	Low-viral loads	Primary monocytes
	GSM361193	Monocyte_LVL_021	Low-viral loads	Primary monocytes
	GSM361198	Monocyte_LVL_028	Low-viral loads	Primary monocytes
	GSM361200	Monocyte_LVL_030	Low-viral loads	Primary monocytes
	GSM361202	Monocyte_LVL_032	Low-viral loads	Primary monocytes
	GSM361212	Monocyte_LVL_043	Low-viral loads	Primary monocytes
	GSM361214	Monocyte_LVL_046	Low-viral loads	Primary monocytes
	GSM361215	Monocyte_LVL_048	Low-viral loads	Primary monocytes
	GSM361217	Monocyte_LVL_050	Low-viral loads	Primary monocytes
	GSM361218	Monocyte_LVL_051	Low-viral loads	Primary monocytes
	GSM361222	Monocyte_LVL_057	Low-viral loads	Primary monocytes
	GSM361230	Monocyte_LVL_067	Low-viral loads	Primary monocytes
	GSM361176	Monocyte_HVL*_001	High-viral loads	Primary monocytes
	GSM361178	Monocyte_HVL_003	High-viral loads	Primary monocytes
	GSM361183	Monocyte_HVL_008	High-viral loads	Primary monocytes
	GSM361184	Monocyte_HVL_009	High-viral loads	Primary monocytes
	GSM361185	Monocyte_HVL_012	High-viral loads	Primary monocytes
	GSM361187	Monocyte_HVL_014	High-viral loads	Primary monocytes
	GSM361189	Monocyte_HVL_016	High-viral loads	Primary monocytes
	GSM361190	Monocyte_HVL_017	High-viral loads	Primary monocytes
	GSM361191	Monocyte_HVL_019	High-viral loads	Primary monocytes
	GSM361192	Monocyte_HVL_020	High-viral loads	Primary monocytes
	GSM361194	Monocyte_HVL_022	High-viral loads	Primary monocytes
	GSM361195	Monocyte_HVL_025	High-viral loads	Primary monocytes
	GSM361196	Monocyte_HVL_026	High-viral loads	Primary monocytes
	GSM361203	Monocyte_HVL_034	High-viral loads	Primary monocytes
	GSM361205	Monocyte_HVL_036	High-viral loads	Primary monocytes
	GSM361208	Monocyte_HVL_039	High-viral loads	Primary monocytes
	GSM361209	Monocyte_HVL_040	High-viral loads	Primary monocytes
	GSM361210	Monocyte_HVL_041	High-viral loads	Primary monocytes
	GSM361219	Monocyte_HVL_053	High-viral loads	Primary monocytes
	GSM361220	Monocyte_HVL_054	High-viral loads	Primary monocytes
	GSM361221	Monocyte_HVL_056	High-viral loads	Primary monocytes
	GSM361223	Monocyte_HVL_058	High-viral loads	Primary monocytes
	GSM361188	Monocyte_C_015	HIV Negative	Primary monocytes
	GSM361197	Monocyte_C_027	HIV Negative	Primary monocytes
	GSM361199	Monocyte_C_029	HIV Negative	Primary monocytes
	GSM361201	Monocyte_C_031	HIV Negative	Primary monocytes
	GSM361204	Monocyte_C_035	HIV Negative	Primary monocytes
	GSM361206	Monocyte_C_037	HIV Negative	Primary monocytes
	GSM361207	Monocyte_C_038	HIV Negative	Primary monocytes
	GSM361211	Monocyte_C_042	HIV Negative	Primary monocytes
	GSM361213	Monocyte_C_044	HIV Negative	Primary monocytes
	GSM361226	Monocyte_C_061	HIV Negative	Primary monocytes
	GSM361228	Monocyte_C_063	HIV Negative	Primary monocytes

*LVL and HVL were renamed to ART-suppressed and NC-TP, respectively in our study.
